# Does ozone gel enhance the bone width and buccal plate of bone thickness surrounding the implant following osseodensification? A randomized controlled clinical trial

**DOI:** 10.1007/s10006-025-01367-x

**Published:** 2025-04-14

**Authors:** Dina Yousry, Raafat Riad, Rehab A. Soliman, Mohamed ElSholkamy

**Affiliations:** 1 Oral & Maxillofacial Surgery Department, Faculty of Dentistry, Misr International University, Cairo, Egypt; 2 Oral & Maxillofacial Surgery Department, Faculty of Dentistry, Suez Canal University, Ismailia, Egypt

**Keywords:** Biomaterials, Closed sinus lifting, Bone augmentation, Transcrestal sinus floor elevation, Osteotomes, CBCT imaging, Clinical research, Simultaneous implants, Implantology, Maxillary sinus floor elevation

## Abstract

**Purpose:**

The present study was conducted to evaluate the effect of using ozone gel with transcrestal sinus elevation using osteotomes, on the bone width and the buccal plate of bone thickness around the implants placed simultaneously.

**Materials and methods:**

A total of 30 sinuses with an average residual alveolar bone height ranging from 4–7 mm participated in this randomized controlled clinical trial. Following a closed sinus lift procedure, patients were randomized into two groups: one for the ozone gel recipient group and the other for control group. Cone beam computed tomography was carried out both immediately and four months postoperatively. Radiographic evaluations were performed to assess bone width and labial plate thickness at both crestal and midcrestal levels.

**Results:**

Radiographic analysis revealed that the mean bone width of the control group after four months postoperative was (8.54 ± 1.46 mm) compared to (8.96 ± 1.66 mm) in the study group, which was statistically insignificant (*P* ≤ .0.05). The mean labial plate of bone thickness value of the control group after four months postoperative was (1.86 ± 0.63 mm) compared to (1.89 ± 0.51 mm) in the study group. Although the bone dimensions in the study group was higher than the control group, it was statistically insignificant (*P* ≤ .0.05).

**Conclusion:**

When compared to the graftless group, the ozone gel recipient group showed non-significant difference in the results in terms of bone width and thickness. Both methods, nevertheless, produced outcomes that were acceptable.

**Trial registration:**

This study protocol was retrospectively registered on the trial registry “Clinical trials.gov PRS”. ClinicalTrials.gov ID is: NCT06604819 and the registration date is 20/9/2024.

**Supplementary Information:**

The online version contains supplementary material available at 10.1007/s10006-025-01367-x.

## Introduction

Partial or total edentulism is one of the major problems in restorative dentistry. Fixed & Removable prosthesis are considered the solutions in some cases but not in all cases. Free end saddle cases represent a challenging situation due to the absence of distal support. Moreover, removable prosthesis is not able to be satisfactory for the patient in terms of stability and function as fixed prosthesis. This situation dictates the need for dental implants, which is one of the successful and predictable treatment procedures to replace the lost teeth [28].

However, due to the horizontal or vertical alveolar ridge deficit, poor bone quality, and maxillary sinus pneumatization, dental implant placement in the edentulous posterior maxilla may be limited. For implant dentistry, the posterior maxilla has been identified as the most challenging and problematic intraoral region, necessitating the highest level of care to ensure a successful procedure [20].

Pneumatized maxillary sinuses are regarded as a relative contraindication to implant placement in the posterior maxillary segments, unless they are treated with prior surgical procedures. The edentulous posterior maxilla has led to the development of several techniques to address bone deficiency. These techniques can be classified as non-surgical (such as short implants or tilted implants) or surgical (such as sinus lift procedures, both open and closed). It has been suggested that a minimum implant length of 10 mm is required to ensure the long-term success of implants, particularly in the maxilla where the bone quality is typically lower than in the mandible, due to high failure rates of short implants (8 mm or less) placed in the posterior maxilla. Consequently, Maxillary sinus floor elevation represents a surgical procedure to vertically enhance the available bone, thus allowing implant positioning with an adequate length in the edentulous posterior maxilla [18, 22, 26].

The sinus lift procedure, or subantral augmentation, was first presented in 1977 and published in 1980 for the purpose of increasing bone quantity within the posterior maxilla [5]. Maxillary sinus lifting technique can be accessed through from four different regions to the sinus It can be approached from Superior-lateral wall of the sinus (Caldwell-Luc), Mid-lateral wall which lies between alveolar crest and zygomatic arch, Inferior-lateral wall which is entered from alveolar crest level (Lateral window technique/external lifting) and last but not least crestal osteotomy (closed technique/internal lifting [32].

The method of osteotome sinus mediated transcrestal lift was originally proposed by Tatum in 1986. In the original techniques, a controlled fracture was done then implants were placed and submerged during the healing phase [32]. Summers proposed a procedure in 1994 that allowed for implant insertion and elevation of the sinus floor from a crestal approach using an osteotome [28].

The author has proposed preparing the implant site with conical osteotomes which allow for compression through lateral force application of bone in the posterior maxilla [27]. According to the author, these maneuvers improve the course of treatment and are less intrusive [1].

The minimal bone height of 4 to 6 mm required to establish sufficient primary stability is one of the technique's initial drawbacks. However, Dental implants' macroscopic design evolved over time, tending toward a conical connection and taking on a conical shape with a reduced thread-to-thread spacing. In regions with limited bone availability, these modifications can lead to increased primary stability [30, 34]. Furthermore, research has surfaced in which implants were positioned in the maxillary region's posterior area in instances with ≤ 4 mm of bone height using a crestal approach, and no statistically significant differences from the traditional procedure were observed [15].

Furthermore, osteotomes are thought to be one of the osseodensification approaches that can help prevent buccal bone abnormalities surrounding implants during implant placement. The technique for osseodensification is a paradigm shift in the way bone tissue is prepared before implants are placed. It has demonstrated encouraging osseointegration outcomes, allowing for the enhancement of bone density at the prepared implant site, circumventing the need for more invasive methods to raise the maxillary sinus membrane, and augmenting the ridge's volume to avert the development of peri-implant bone defects [14].

After a tooth is extracted, the alveolar process remodels and promotes ridge morphology alterations that may make implant placement more difficult or impossible without prior bone grafting. Bone grafts have been widely utilized to address minor peri-implant deficiencies during implant installation or to provide adequate anchoring before implant placement. Bone grafting is associated with increased treatment costs and duration, surgical morbidity, and postoperative medicine usage, while being well-documented as having high predictability. Additionally, patient acceptance is negatively impacted [33].

Autogenous bone is regarded as the gold standard for bone grafting because of its osteogenicity, osteoinductivity, and osteoconductivity [35]. However, different options were searched for because of the substantial graft resorption rate and donor site morbidity [23, 24, 39]. A low vital bone to biomaterial ratio, a slow rate of resorption, and the transmission of disease has all been linked to alternative bone substitutes like synthetic alloplast, human allograft, and bovine xenograft. Furthermore, in comparison to autogenous bone or blood clot alone, they may postpone bone regeneration [8].

As a result, the use of biological augmentations evolved. For more than a century, medical-grade ozone has been used as a non-pharmaceutical treatment technique. In Germany, ozone therapy was first used as a state-of-the-art remedy for wounds and infections in the 1950s. These days, there is growing evidence that it can be applied as a helpful therapeutic agent in the fields of medicine and dentistry. In the 1930s, E. A. Fisch was the first dentist to use ozone therapy in his office to encourage wound healing and disinfection. Modern medical ozone generators regulate the flow of medical-grade oxygen through high-voltage tubes to produce precise dosages of pure ozone-oxygen mixes. Pure oxygen and ozone gel are combined to create classical ozone [4, 29] Ozone can be applied in three forms which are ozonated water, ozonated oil or gel, and oxygen/ozone gas. When ozone comes into contact with a lesion, ozone-entrapped oil or gel will release its trapped ions [6].

As a super-oxygenator, ozone helps the body's natural healing processes by delivering oxygen to tissues. O3 works by inducing growth factor release and promoting hemostasis. Additionally, it can boost immunological responses and blood circulation [9, 25]. To reduce inflammation and discomfort, biologically active chemicals including interleukins, leukotrienes, and prostaglandins are synthesized with the help of ozone. Ozone, then, is widely recognized for having analgesic and anti-inflammatory qualities [16, 31].

Because of its potent bactericidal, fungicidal, virostatic, and immune-stimulating capabilities, ozone treatment has demonstrated therapeutic efficacy in treating diseases caused by bacteria, fungi, and viruses. Furthermore, it has been reported that ozone therapy promotes healing, aids in bone remineralization, increases tissue oxygenation and density, boosts antioxidant defenses directly, sanitizes and sterilizes infected wounds, and reduces excessive humoral "antibody" activity [7, 11]. The peri-implant interface zone is thought to have been improved by ozone therapy's bactericidal activity, local oxygen supply enhancement, and hemostasis promotion. This may have resulted in an increase in osteoblast proliferation, which in turn increased the rate and volume of bone formation and mineralization on the peri-implant bone interface [19]. Ozone therapy has a therapeutic effect that promotes blood and growth factor supply, aids in wound healing, and may improve bone regeneration [10].

This was further demonstrated by a different study that evaluated the viability of autogenous bone grafts taken from the calvaria of rabbits after the grafts had been decontaminated with three distinct antibacterial agents. Twenty-four white rabbits' calvaria were used to gather data on the effects of autogenous bone grafting. The grafts were exposed to 2% chlorhexidine (Group II), clindamycine (Group III), oleozone gel (Group IV), and normal saline (Group I) for a duration of five minutes each.

Histomorphometric analysis showed that the group that received ozone treatment had a better result, as evidenced by a noticeably greater proportion of normal osteocytes and a notable rise in the area percentage of new bone formation. Osteoblastic vitality was notably preserved in the ozone-treated grafts [17].

This was supported by previous study, which concluded that ozone therapy has a positive impact on bone metabolism and improvement of reparative process of bone. Significant decrease in healing time and defects treated with ozonated gel were reported. Another animal study evaluating the use of ozonated gel in mandibular defected reported by histological analysis better vascularity and bone density in the study group which received the ozonated gel in the mandibular defect [21, 29].

Nevertheless, there is not enough evidence to support the use of ozone in oral and maxillofacial surgery. However up till the date of publication, no studies were conducted to assess the impact of using ozone gel on bone formation in case of maxillary sinus elevation. Therefore, the present study was conducted to evaluate the effect of using ozone gel with transcrestal sinus elevation using osteotomes, on the bone width and the labial plate of bone thickness around the implants placed simultaneously.

## Materials and methods

In all, 19 patients (12 men and 7 women) participated in this randomized controlled clinical study. Patients were chosen from the Suez Canal University Faculty of Dentistry Oral and Maxillofacial Department outpatient clinic. Each patient in this study has sinus pneumatization and was seeking fixed prosthetic rehabilitation in the maxillary posterior region. G* Power version 3.1.9.4 was used to calculate the sample size based on prior research. A two-sided hypothesis test was used with an adjusted sample size of 30 (15 in each group) to detect an effective size of 1.10 and power (1-B) of 0.8 to account for an expected loss to follow-up. Alpha error (significance level) for the data is set at 0.05 [13, 22]. The ethics committee of Suez Canal University's faculty of dentistry approved this parallel study, and the treating dentists notified the patients of its goal and methodology. The individuals were progressively added between December 2021 and December 2023. The informed consent was signed by every patient.

**Fig. 1:**
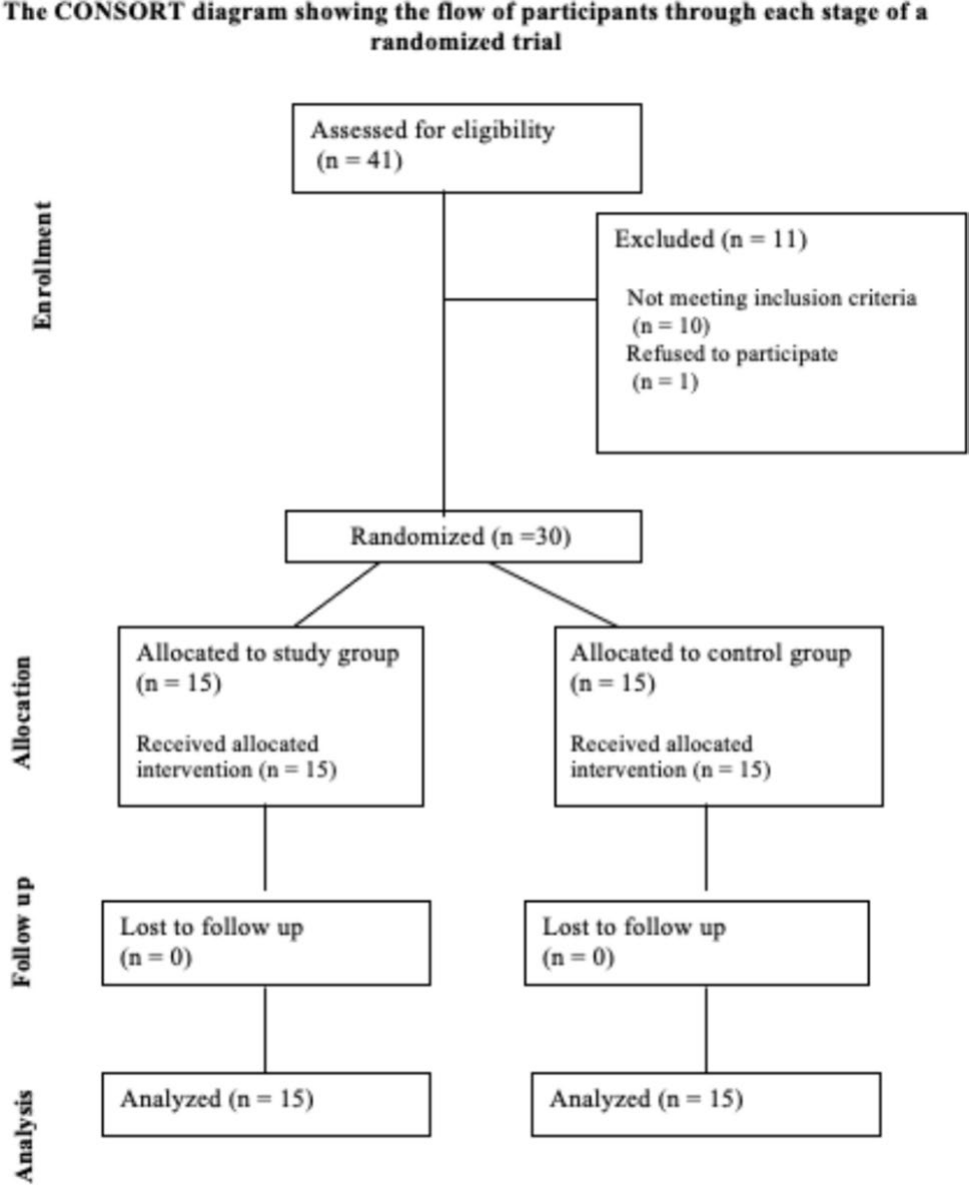
The consort flowchart

The clinical trial investigator created a basic random allocation sequence using an open generator (randomizer.org). The supervisor of the outpatient clinic at Suez Canal University's Faculty of Dentistry, Oral and Maxillofacial Departments, recruited patients. The trial's primary supervisor assigned the participants to the intervention once the principal investigator had completed their enrollment. The results were evaluated by the other two co-supervisors.The patients who were chosen had multiple tooth defect with residual alveolar bone heights between 4 and 7 mm (Table 1). They were also free of systemic illnesses and medications that can impede natural bone healing or the osseointegration of dental implants. Individuals who smoked more than 20 cigarettes a day, had disease in their surrounding teeth, had residual roots pushed into their sinuses, or had maxillary sinus pathosis were denied the opportunity to participate in the study (Fig. 1). This study adheres to CONSORT guidelines.

**Table 1:** Measurements of amount of preoperative residual bone height

Sinus no	Residual bone height (ozone group)	Residual bone height (control group)
1	4 mm	7
2	5 mm	7
3	6	6.9
4	6.1	5.81
5	6.73	4.69
6	5.16	5.7
7	6.1	6.7
8	5.2	6.5
9	6.7	5
10	6.7	7
11	5	6.5
12	6	4.2
13	5	6.6
14	5.5	7
15	5.6	6.9

### Preoperative preparation

**Fig. 2:**
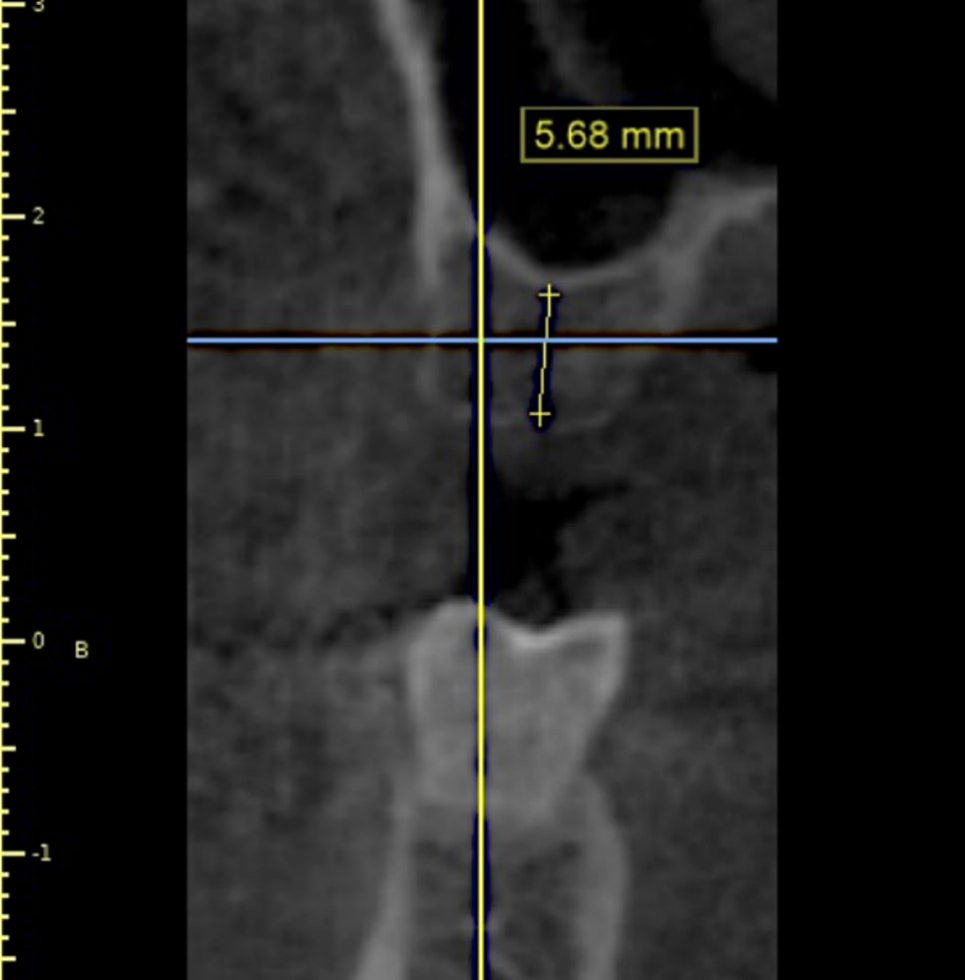
Sections of reformatted sagittal cut from the preoperative CBCT to assess presence of any sinus pathosis and to accurately measure the residual alveolar bone height

Every patient who was enrolled in the research underwent a radiographic analysis following a clinical evaluation. For each patient, a cone beam computed tomography (CBCT) of the maxilla was ordered in order to precisely measure the amount of residual alveolar bone height and determine whether any sinus pathosis was present. The width and length of the implants that needed to be placed were calculated using the CBCT's reconstructed images (Fig. 2). For the patients, both intraoral and extraoral photographs were taken.

### Surgical procedure

Two groups of thirty sinuses each were randomly assigned (15 in each group). Randomizer.org, an open generator, was used to create a random allocation sequence. As a result, each participant had an equal chance of being allocated to the control group or the study group. In Group (A), ozone gel was used concurrently with implant insertion, after elevating the sinus membrane using osteotomes. Group (B) underwent graftless sinus membrane elevation using osteotomes concurrent with implant insertion.

**Fig. 3:**
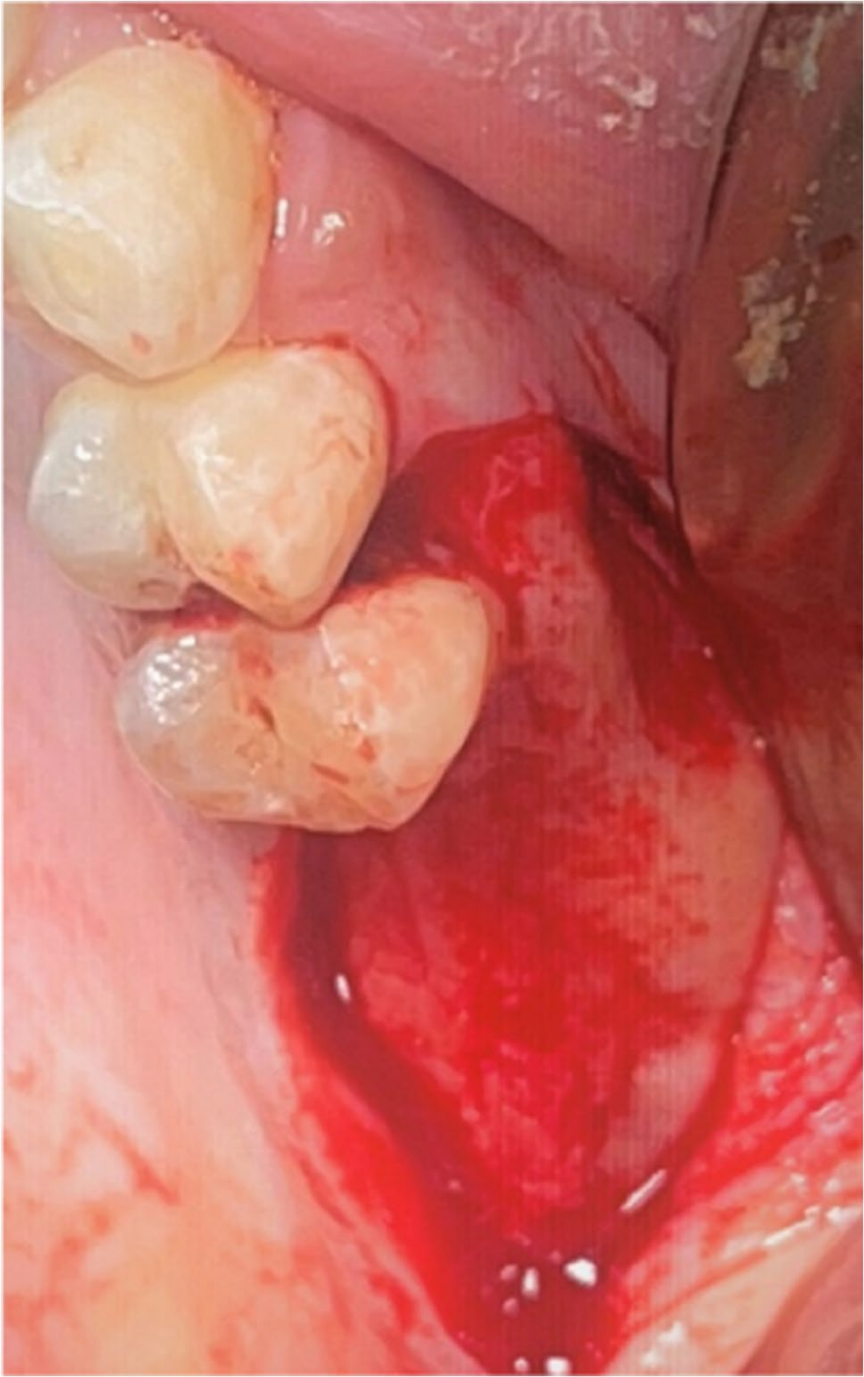
Full thickness mucoperiosteal flap

Every patient underwent the identical surgical procedure. The patients were instructed to rinse their mouths twice a day for a week with an antiseptic mouthwash containing 0.2% chlorhexidine (Orovex Mouthwash, Macro Group, Egypt) prior to surgery approximately two minutes before the surgical operation. After injecting adrenaline 1:100,000 and Articaine 4% as local anesthetic, a full thickness mucoperiosteal flap was elevated during the procedure. A full thickness mucoperiosteal flap was elevated by starting a mid-crestal incision with a no. 15 blade and a mesial vertical releasing incision (Fig. 3).

**Fig. 4:**
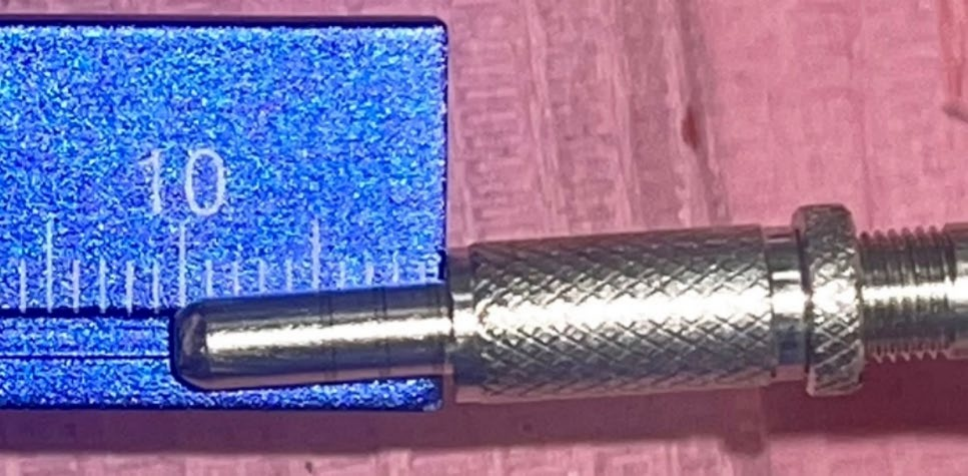
Penetration height of the osteotome was determined

**Fig. 5:**
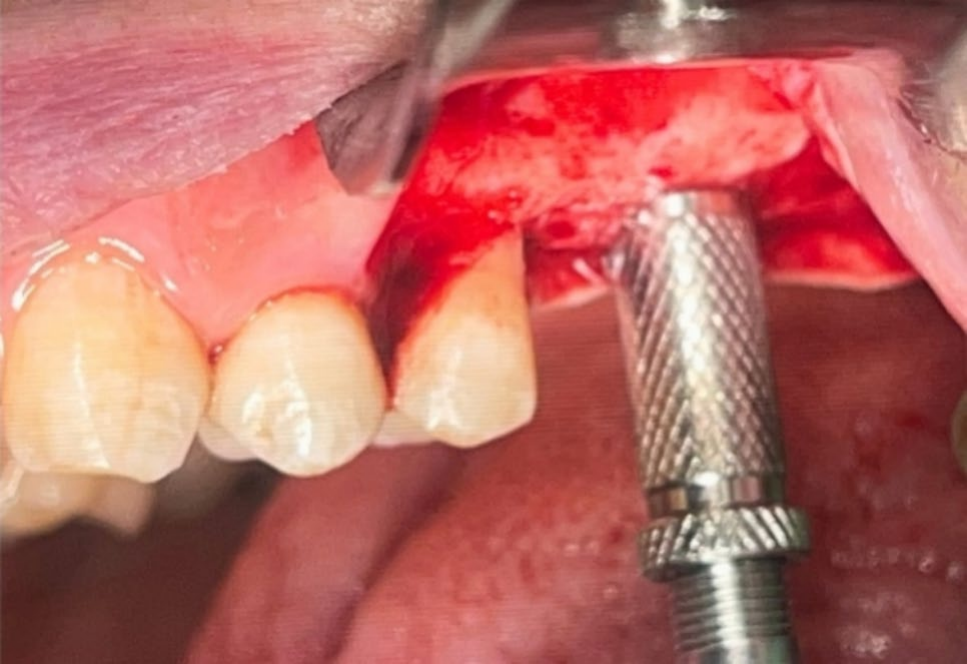
Sequential use of osteotomes

**Fig. 6:**
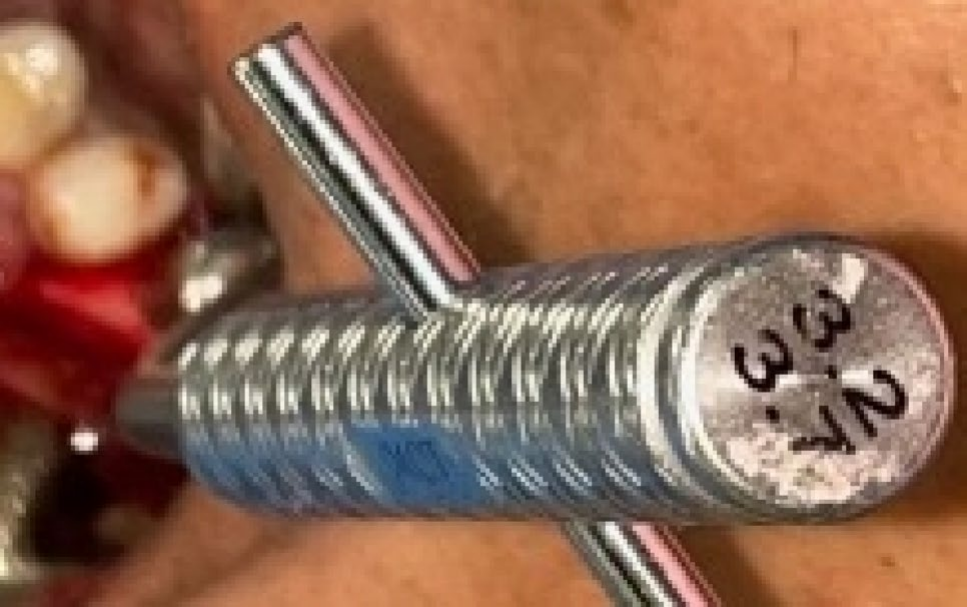
The last osteotome corresponding in size to the last drill that should be used

An osteotomy was made using the implant system's pilot drill (Nebiotech, Seoul, South Korea; ) 1 mm below the subantral floor. Drill penetration was halted when the drill was carefully pushed through the cancellous bone in an apical orientation until the cortical bone resistance of the sinus floor was reached. A metal stopper was inserted on the osteotome before use in order to keep it from entering the sinus (Fig. 4). The osteotome was used to compress the sinus membrane in an apical direction with very little pressure, rotation, and, if needed, gentle malleting. Until the necessary membrane lift was obtained, the osteotome insertion was performed multiple times (Fig. 5). Next, the osteotome that was the same size as the last drill that should be utilized, which was smaller than the implant to be placed, was employed to up- fracture the sinus floor (Fig. 6).

**Fig. 7:**
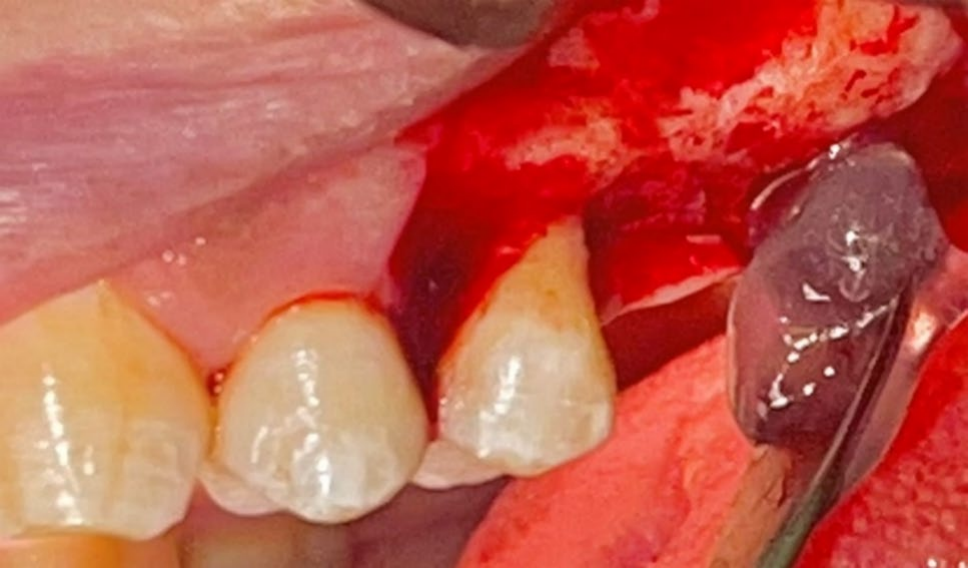
Ozone gel delivered into the osteotomy

**Fig. 8:**
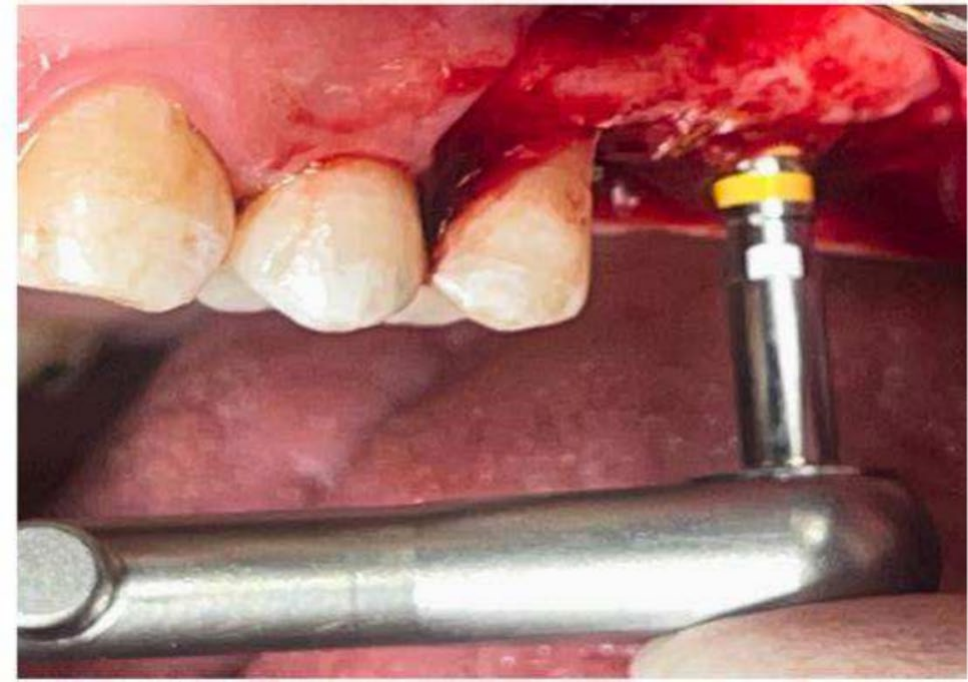
Implant placement done

Ozone gel was injected into the osteotomy for group (A) (Fig. 7). Pure olive oil was exposed to 25 μ/ml O3 gas for two days, or until the oil turned from a greenish-colored liquid to a whitish gel, in order to create the ozone gel. This was done using the longevity Ext 120 ozone generator. The implant was then placed after that. In group (B), however, the implant was placed and no graft material was added to the sinus cavity (a technique known as graftless tenting) (Fig. 8).

### Postoperative instructions and medication

For seven days following surgery Antibiotics were prescribed Amoxicillin/ Clavulanic acid (1)875mg/125mg oral tablet twice daily for 7 days postoperatively (or Clindamycin 300 mg) in patients whom allergic to penicillin 3 times daily). 600 mg of Ibuprofen was prescribed for three days of postoperative analgesia, or as needed for pain relief, every eight hours. For four days, apply two drops of the nasal decongestant (oxymetazoline 0.25%) to each nostril every six hours.

–Patients were advised against blowing their noses, to sneeze with their mouths open, and to avoid bearing down when performing tasks that increase nasal or oral pressure, such as lifting heavy objects, blowing up balloons, playing musical instruments, or doing anything else that involves blowing.

**Fig. 9:**
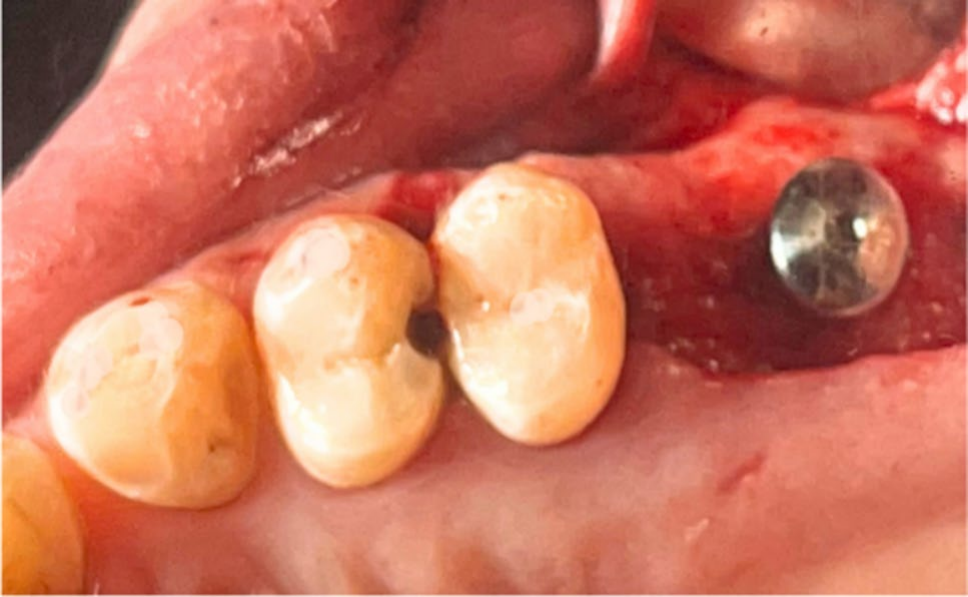
Healing abutment over the implant fixture

**Fig. 10:**
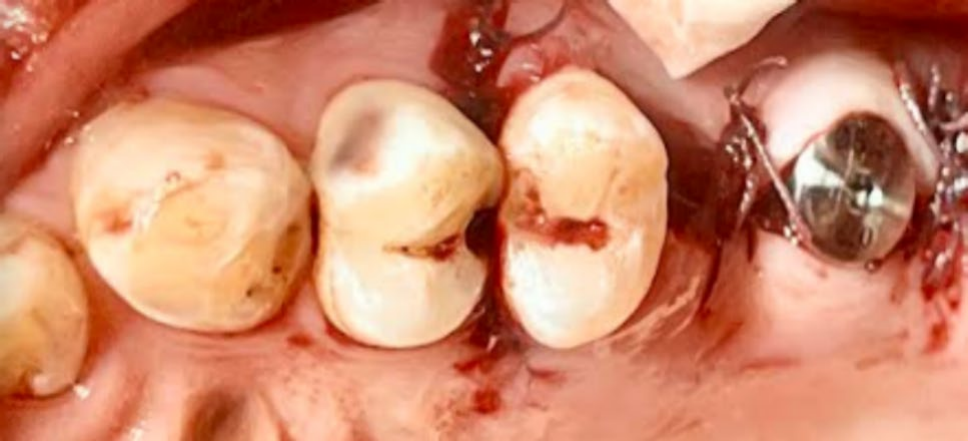
Primary flap closure

In each group, a healing abutment was positioned (Fig. 9) primary closure was carried out, and the flap was interruptedly sutured with 4/0 prolene suture (Fig. 10).

### Postoperative follow-up and assessment

#### Clinical assessment

**Fig. 11:**
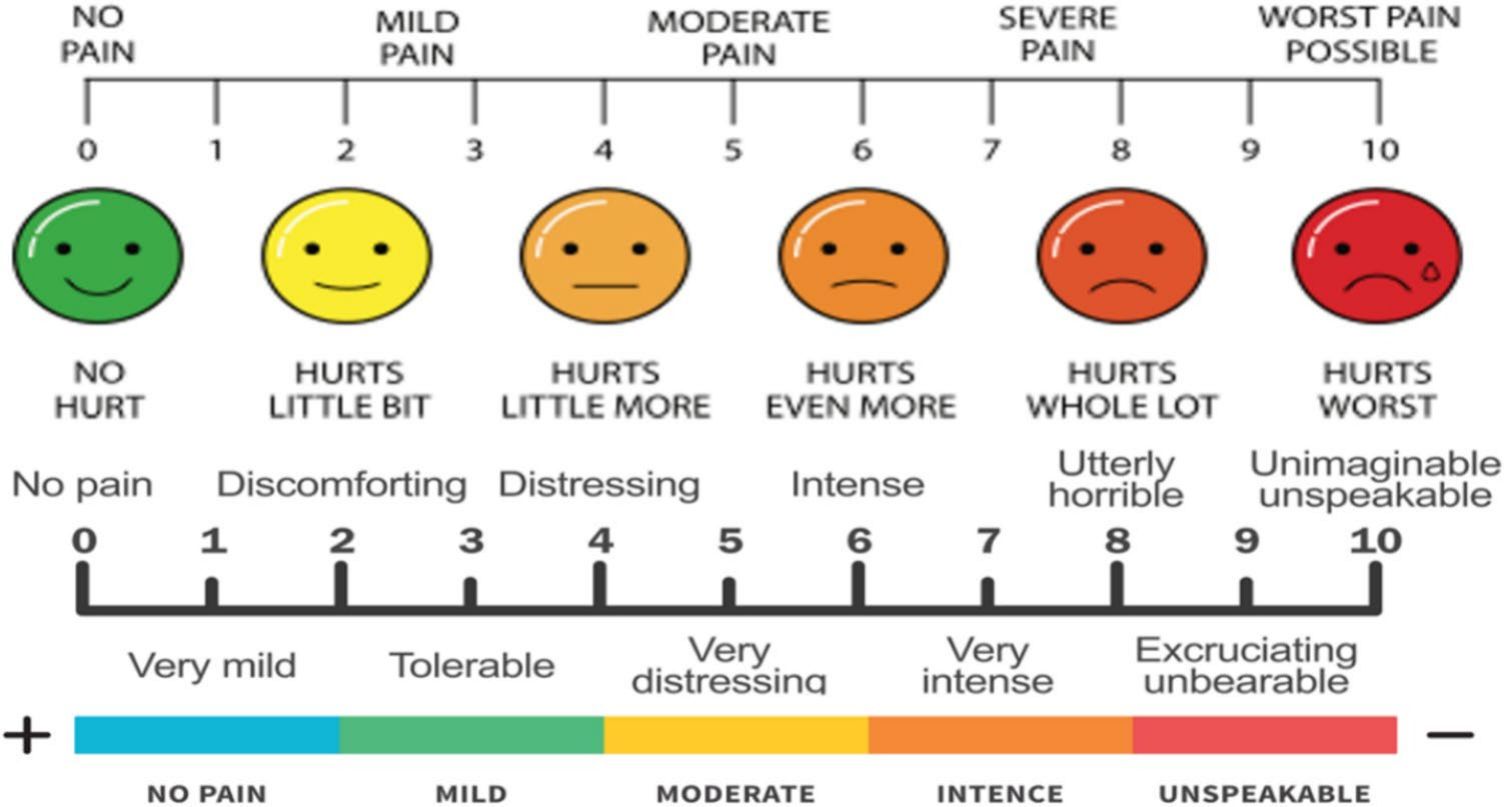
Showing pain visual analogue scale

One week following surgery, patients underwent a clinical assessment to determine whether they experienced pain using the Visual Analogue scale (Fig. 11), as well as any other complications related to implant placement or sinus lifting.

#### Radiographic assessment

**Fig. 12:**
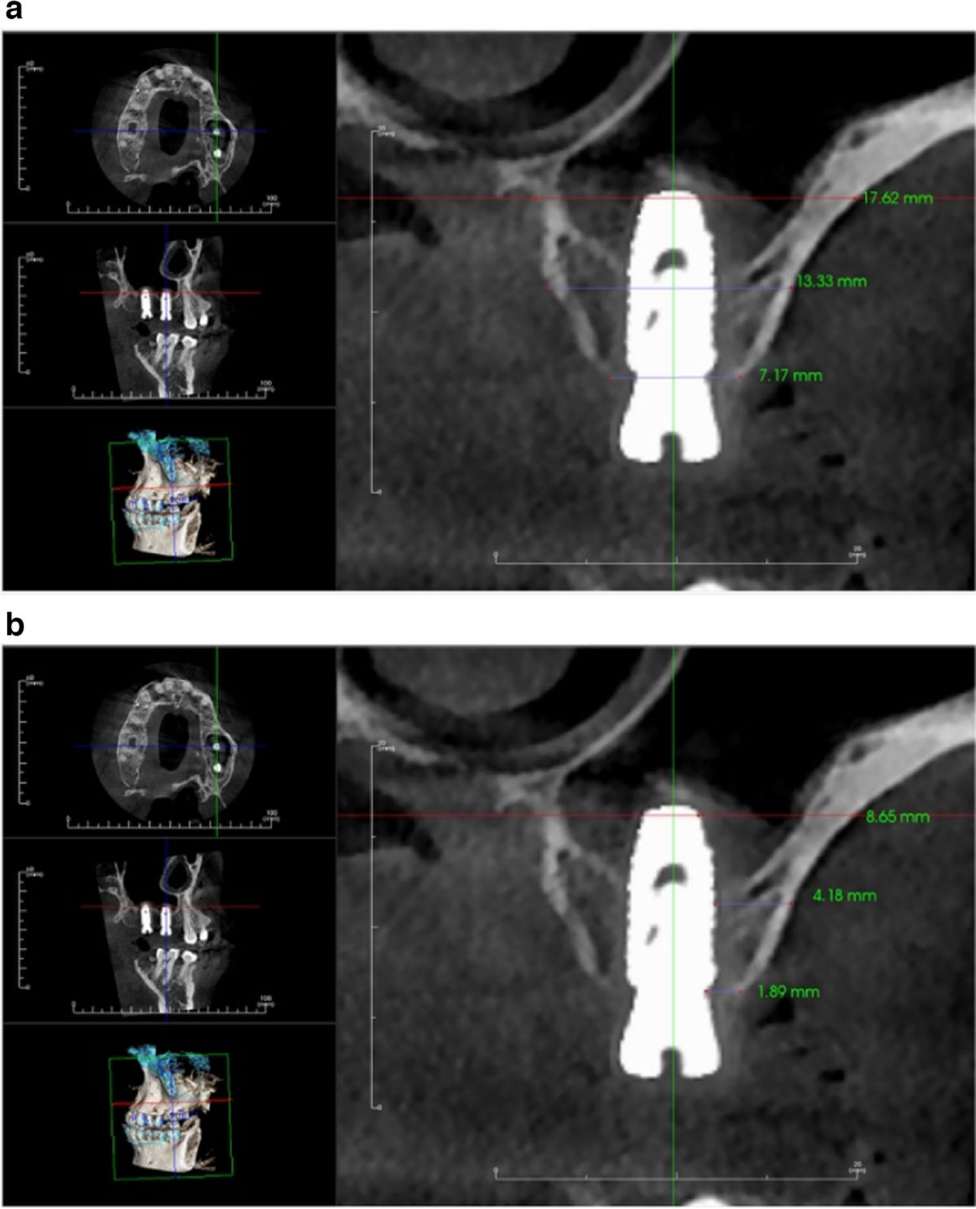
**a** Immediate postoperative CBCT showing bone width. **b** Immediate postoperative CBCT showing labial plate of bone thickness

Immediate postoperative cone beam computed tomography (CBCT) was taken to evaluate the accuracy of implant placement and to represent the baseline measurement for evaluation. Bone width as well as the labial plate of bone thickness was measured at three levels, crestal, mid-crestal and apical (Fig. 12 (a-b)). To standardize the measurements, cross sections from the immediate postoperative CBCT were taken coinciding with the long axis of each implant.

**Fig. 13:**
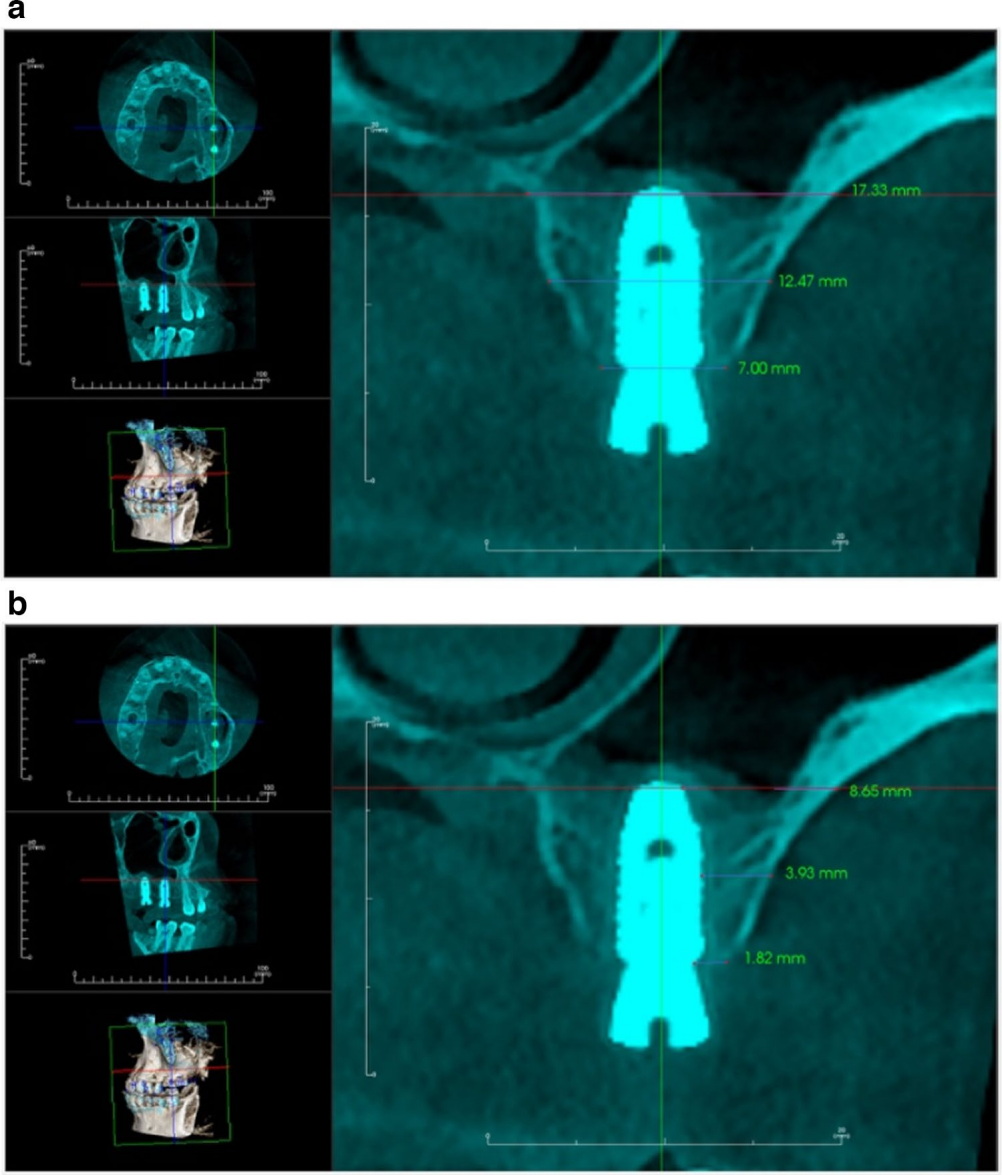
**a** 4 months-postoperative CBCT showing bone width. **b** 4 months- postoperative CBCT showing labial plate of bone thickness

Four months after surgery, a second CBCT was performed to assess postoperative measures and the overall healing process. A cross section was obtained from a 4-month postoperative CBCT that coincided with each implant's long axis (Fig. 13(a-b)) (Table 2). The same equipment and exposure settings (Planmeca, Promax—Finland, 15 mA, 85 KV) were used to create the radiographs.

**Table 2:** Measurements of amount of bone width “crestal & mid-crestal”, and labial plate of bone thickness for all 30 sinuses 4 months postoperative

Sinus no	Average mid-crestal width values (ozone group)	Average crestal width values (ozone group)	Average mid-Apical width values (ozone group)	Average mid-crestal width values (control group)	Average crestal width values (control group)	Average apical width values (control group)	Average mid-crestal labial plate of bone thickness values (ozone group)	Average mid-crestal labial plate of bone thickness values (control group)	Average crestal labial plate of bone thickness values (ozone group)	Average crestal labial plate of bone thickness values (control group)
1	8.5	6.3	10.4	8	6	10.3	1.4	0.7	0.56	0.35
2	10.3	9	15	7.9	5.8	10.6	2.8	1	1.2	0.6
3	8.6	5.9	11.5	10	8	12.4	2	3.7	0.64	1.27
4	10.8	6.7	15	9	8.4	13.2	3.3	3.2	1.62	1.7
5	14	10	17	8.17	7.7	10.25	3	1.7	1.6	1.2
6	10.8	8.9	16.3	13.3	10.2	17	3.9	3.6	1.8	2.15
7	8.6	7	11.7	12	10	16.5	2.21	3	1.92	1.8
8	12.4	8.9	16	9	5.4	13	2.2	3.1	0.92	0.9
9	13	8.2	16.3	8	6	12.3	2.5	2.8	1.4	0.7
10	9.9	6.2	11.5	9	7.3	13.8	3	2.9	1.5	0.8
11	11.1	8.5	15.4	9	7.4	13.8	1.9	2.5	0.9	2
12	13.3	9	17.5	11.3	9.3	16.42	3.2	3	1.1	1.8
13	7.3	7	10	8.7	7.7	12	2.1	1.8	1,14	1.4
14	8.5	6	11	8.9	6	11	0.9	1.9	0.9	1.1
15	7.9	6.4	11.8	8.7	6.4	11.8	1.8	2.4	1.8	1.2

### Bone gain

**Fig. 14:**
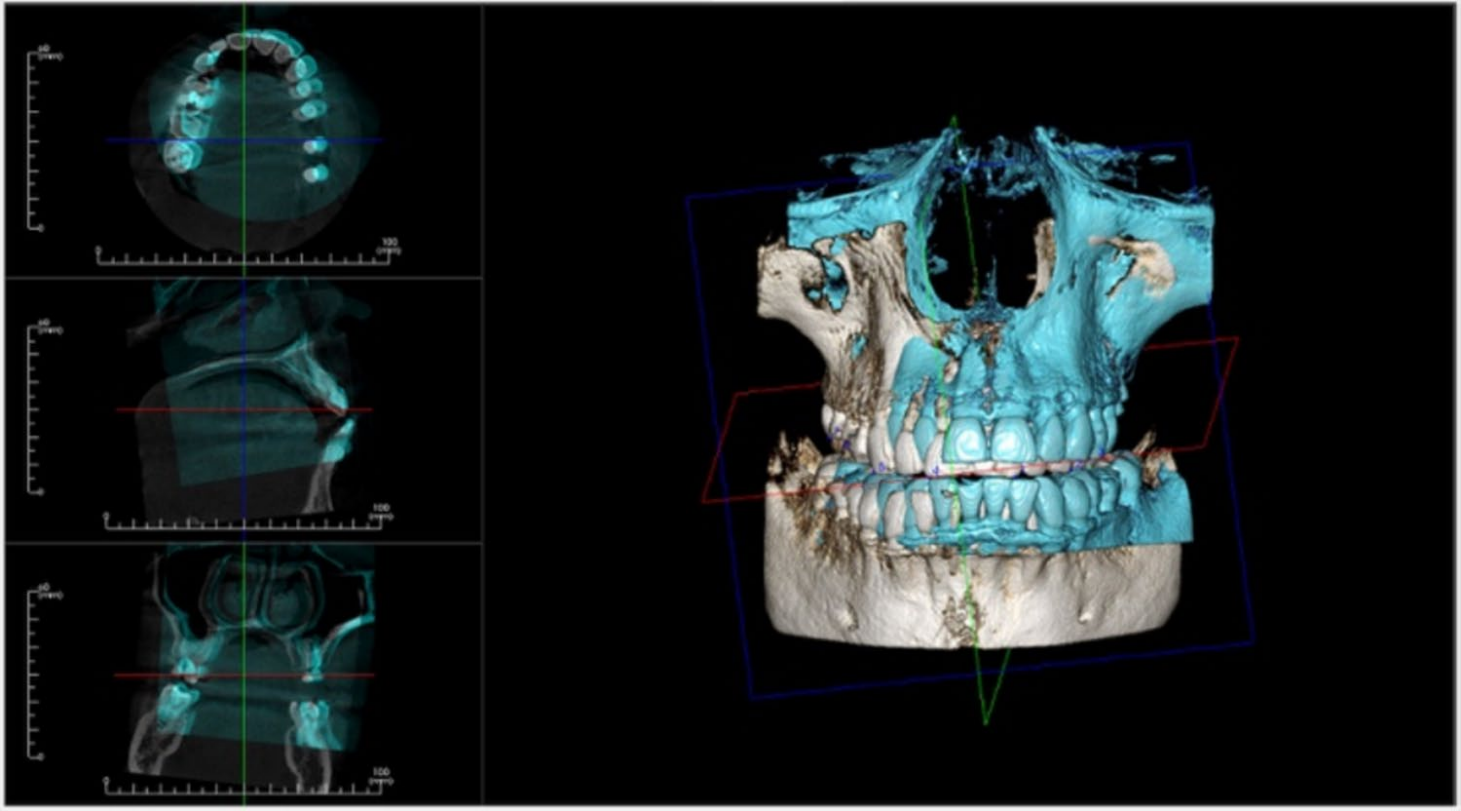
Showing the fusion of CBCTs

To determine the degree of bone change for each implant, the values of the native bone measures were deducted from those obtained from the 4-month CBCT. The On Demand 3D fusion module software (Cybermed Inc., Seoul, South Korea) was utilized to create a superimposed image at the sagittal and coronal planes of each implant in order to compare each parameter and reduce the error from radiography image alignment (Fig. 14).

**Fig. 15:**
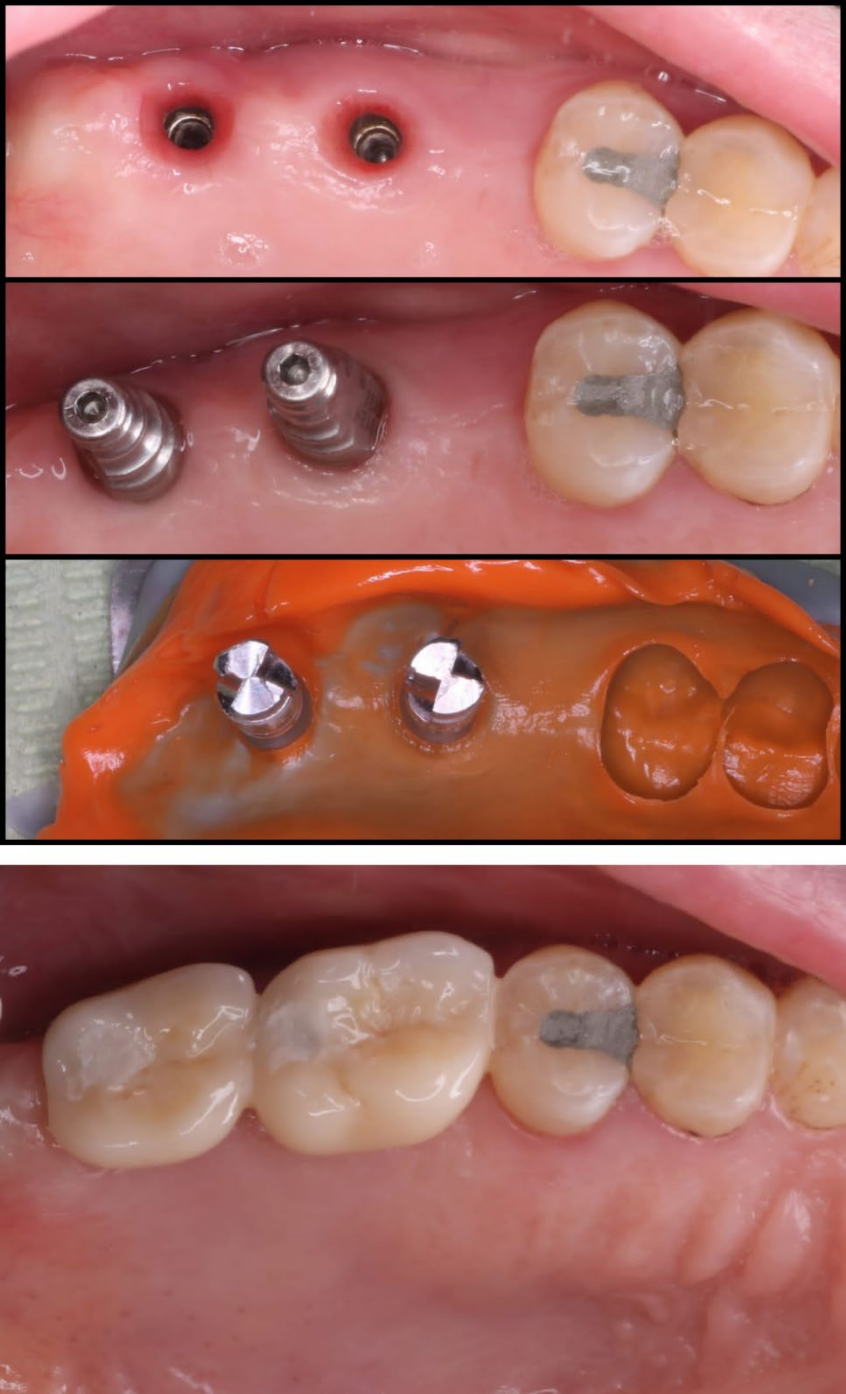
Metal ceramic prosthesis cemented on the implant

As advised by the manufacturer, 35 N/cm of torque was used to secure the prosthetic abutments to the implants. Every implant resisted the torque that was exerted, and metal-ceramic prostheses were manufactured and cemented on the implant using the traditional prosthetic methods (Fig. 15).

### Statistical analysis

By examining the distribution of the data and applying normalcy tests (Kolmogorov–Smirnov and Shapiro–Wilk tests), numerical data were examined for normality. With the exception of changes, % changes in bone height, assessments of bone density, and VAS scores, which are non-parametric data, all data displayed a normal (parametric) distribution. The values of the mean, standard deviation (SD), median, and range were displayed for the data. The repeated measures ANOVA test was employed for parametric data in order to examine changes over time within each group and to compare the two groups. When the ANOVA test is significant, pairwise comparisons were performed using Bonferroni's post-hoc test. The mean ages of the two groups were compared using the student's t-test.

By examining the distribution of the data and applying normalcy tests (Kolmogorov–Smirnov and Shapiro–Wilk tests), numerical data were examined for normality. With the exception of changes, % changes in bone width, assessments of labial plate of bone thickness, and VAS scores, the repeated measures ANOVA test was employed for parametric data in order to examine changes over time within each group and to compare the two groups. When the ANOVA test is significant, pairwise comparisons were performed using Bonferroni's post-hoc test. The mean ages of the two groups were compared using the student's t-test. which are non-parametric data, all data displayed a normal (parametric) distribution. The values of the mean, standard deviation (SD), median, and range were displayed for the data.

## Results

The 19 patients in the study ranged in age from 24 to 59 years old (average 42.9± 4.9 years), with 17 females and 2 males. Both groups received thirty implants. The implants that were inserted ranged in widths from 3.7 to 4.8 mm and in height from 10 to 12 mm (Tables 1 and 3).

**Table 3:** Descriptive statistics and results of Fisher’s exact test and Student’s t-test for comparisons between base line characteristics in the two groups

Baseline characteristics	Study (*n* = 15)	Control (*n* = 15)	*P*-value
Gender [*n*, (%)]			
Male	1 (6.7%)	2 (13.3%)	1
Female	14 (93.3%)	13 (86.7%)	
Age [Mean, SD]	42.9 (4.9)	43.1 (4.5)	0.907
Tooth [*n*, (%)]			
Premolar	3 (20%)	4 (26.7%)	1
Molar	12 (80%)	11 (73.3%)	

### Clinical results

There were no signs of postoperative infection, dehiscence, or oroantral communications in any of the patients, who all healed without incident. All patient data were included for analysis, and no patients were lost for follow-up. During the follow-up period, no implants were lost, and all the implants placed in both groups were successfully loaded and clinically stable at the prosthetic stage. There was no statistically significant difference between pain scores in the two groups (*P*-value = 0.763, Effect size = 0.106).

### Radiographic results

Residual bone height: The preoperative CBCT showed that the average preoperative residual bone height of the control group was (6.23 ± 0.94 mm) compared to (5.65 ± 0.78mm) in the study group, which was statistically insignificant (*P* ≤.0.05) (Table 4).

**Table 4:** Mean, standard deviation (SD) values and results of repeated measures independent t test for comparison between preoperative residual bone height measurements (mm) in the two groups

Time	Study (*n* = 15)		Control (*n* = 15)		*P*-value	Effect size (Partial Eta squared)
	Mean	SD	Mean	SD		
Pre-operative	5.65	0.78	6.23	0.94	0.075	0.109

*: Significant at *P* ≤ 0.05

#### Bone width

**Fig. 16:**
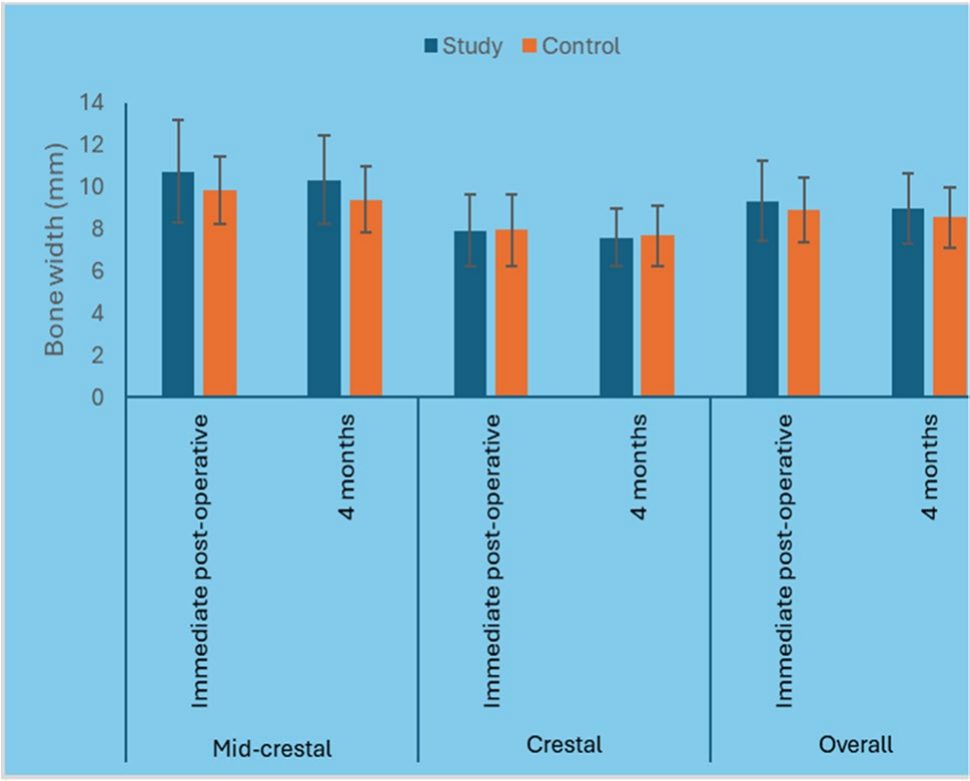
Bar chart representing mean and standard deviation values for bone width measurements in the two groups

The 4 months postoperative CBCT revealed that the mean bone width of the control group after four months postoperative was (8.54 ± 1.46 mm) compared to (8.96 ± 1.66mm) in the study group, which was statistically insignificant (*P* ≤.0.05) (Figure 16).

Bone width change: The mean bone width changes percentage of the control group after four months postoperative was (−2.7 %) compared to (0.4 %) in the study group. However, it was statistically insignificant (*P* ≤.0.05) (Table 5).

**Table 5:** Descriptive statistics and results of Mann–Whitney U test for comparison between bone width change (mm) and percentage of change in the two groups

Level		Study (*n* = 15)		Control (*n* = 15)		*P*-value	Effect size (d)
		Median (Range)	Mean (SD)	Median (Range)	Mean (SD)		
Mid-crestal	Change (mm)	−0.2 (−2.74, 1)	−0.4 (1.15)	−0.43 (−3.77, 1.65)	−0.42 (1.41)	0.868	0.061
	Change (%)	−1.8 (−18.1, 14.5)	−2.7 (10.5)	−5 (−32, 22.5)	−3.2 (13.8)	0.917	0.038
Crestal	Change (mm)	−0.4 (−1.77, 2.24)	−0.34 (1.01)	−0.2 (−1.77, 2.24)	−0.28 (1.02)	0.917	0.038
	Change (%)	−4.3 (−21.3, 30.4)	−2.7 (13.7)	−3.3 (−18.8, 40.7)	−2.3 (14.4)	0.934	0.03
Overall	Change (mm)	−0.43 (−6.98, 5.16)	−1.15 (3.94)	−1.62 (−12.13, 7.89)	−1.31 (5.17)	0.983	0.008
	Change (%)	0.4 (−10, 4.8)	−1.9 (6.2)	−2.7 (−13.9, 12.2)	−1.9 (7.8)	0.950	0.023

#### Bone thickness

**Fig. 17:**
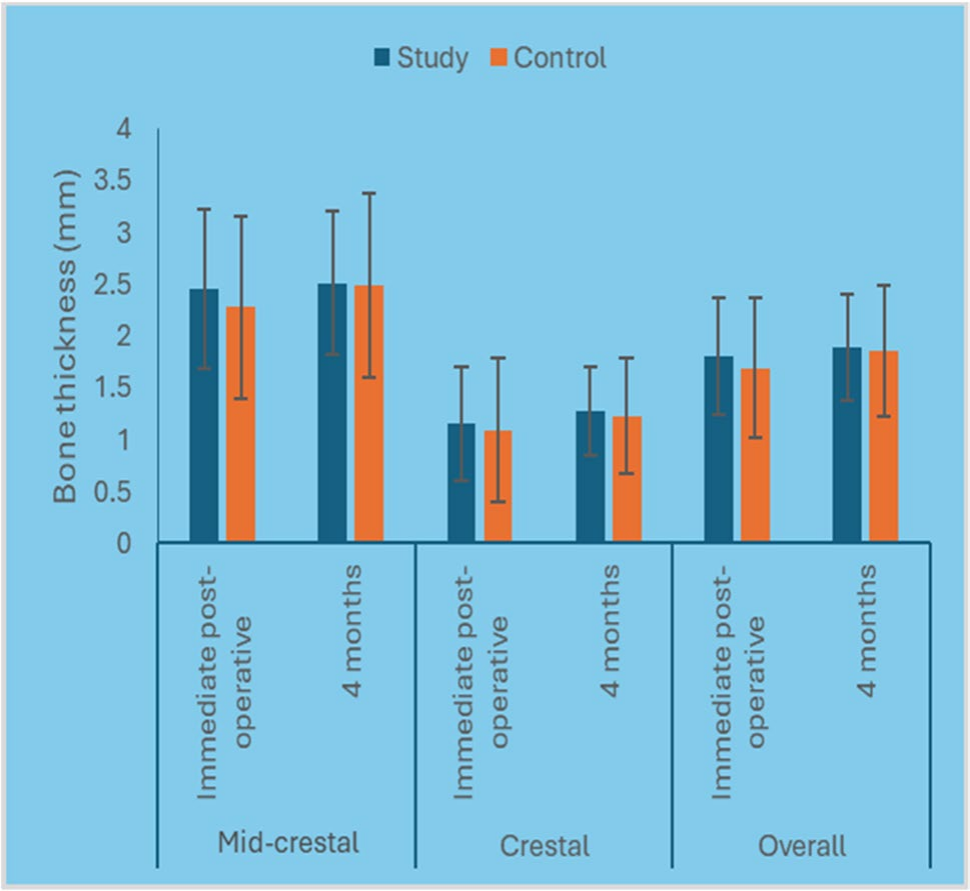
Bar chart representing mean and standard deviation values for bone thickness measurements in the two groups

The mean bone thickness value of the control group after four months postoperative was (1.86 ±0.63) compared to (1.89±0.51 HU) in the study group. Although the bone thickness in the study group was higher than the control group, it was statistically insignificant (*P* ≤.0.05) (Fig. 17).

#### Bone thickness change

The mean bone thickness change percentage of the control group after four months postoperative was (3.4 %) compared to (6.3 %) in the study group. However, it was statistically insignificant (*P* ≤.0.05) (Table 6).

**Table 6:** Descriptive statistics and results of Mann–Whitney U test for comparison between bone thickness change (mm) and percentage of change in the two groups

Level		Study (*n* = 15)		Control (*n* = 15)		*P*-value	Effect size (d)
		Median (Range)	Mean (SD)	Median (Range)	Mean (SD)		
Mid-crestal	Change (mm)	0.19 (−1.5, 1.19)	0.05 (0.74)	0.07 (−0.49, 1.2)	0.2 (0.47)	0.983	0.008
	Change (%)	8.7 (−40.2, 73.9)	8.2 (33.1)	2.47 (−33.3, 119.5)	13.5 (37.1)	0.983	0.008
Crestal	Change (mm)	0.1 (−0.84, 1.06)	0.12 (0.51)	0.16 (−0.66, 1.19)	0.14 (0.46)	0.983	0.008
	Change (%)	12.5 (−60, 220)	39.1 (84.3)	16.3 (−33.3, 195.1)	32.3 (63.4)	0.917	0.038
Overall	Change (mm)	0.1 (−0.88, 1.01)	0.09 (0.56)	0.03 (−0.3, 1.09)	0.17 (0.43)	0.885	0.053
	Change (%)	6.3 (−49.3, 144.9)	23.6 (54.3)	3.4 (−23.2, 157.3)	22.9 (46.9)	0.983	0.008

## Discussion

Whether the addition of bone graft material for indirect sinus augmentation enhances bone development is yet unknown due to the many confounding factors that affect implant lifespan. In patients with atrophied posterior maxilla, the transalveolar osteotome technique has been demonstrated to be a dependable therapeutic option for establishing adequate bone height for dental implant placement. Nevertheless, other techniques were created to enhance surgical results by avoiding common problems related to the conventional sinus elevation method. In addition to implant placement, Summers suggested a method that enabled the sinus floor to be raised from crestal access using an osteotome [36, 37].

Therefore, the goal of the present research was to evaluate the effect of using ozone gel with transcrestal sinus elevation using osteotomes, on the bone width and the labial plate of bone thickness around the implants placed simultaneously.The patients in this study were older than 18 years old. The distribution of teeth in the two groups did not differ statistically significantly based on gender. In terms of gender, the percentage of females was 90% higher than that of males (10:1), which could be explained by the fact that women are more interested in replacing their teeth for both functional and aesthetic reasons.

The two groups' pain scores did not differ statistically significantly (*P*-value = 0.763, Effect size = 0.106).This outcome was consistent with multiple studies conducted by Taschieri et al. in 2017 and 2018, which verified that the crestal sinus lift approach resulted in significantly better postoperative healing (e.g., less inflammation, less pain, and faster recovery including normal daily activities) and lower patient morbidity because less extensive mucoperiosteal flaps were needed when compared to the lateral window procedure [38].

Regarding the surgical procedure, the under-drilling technique used allows adequate osteointegration for the implants placed after 4 months and this was in accordance with the results of a study conducted by Andrés-García and his colleagues. They evaluated the survival of 32 implants placed in posterior maxilla with bone availability less than 5 mm performing a sinus lift augmentation technique with osteotome without biomaterials where spontaneous bone formation was observed in all the cases after 12 weeks [2]. However, in that study [2], the initial available bone was from 2–5 mm and not from 4–7 mm as in our study which clarifies that this proposed technique reduces treatment time and the need for more invasive maxillary sinus augmentation techniques.

The results of our study revealed that there was no significant difference in the overall width values “crestal, mid-crestal and apical “values between both groups. These results were not competent with the results obtained from another study published by Frizzera et al. in 2022 where there was significant increase in the ridge width. However, this study was conducted on pig mandibles not posterior maxilla where densa burs were used for osseodensification instead of osteotomes.Morevover in our study osseodensification was used in both groups unlike Frizzera study where Densa burs were compared to the normal implant drilling, explained by the findings of Fanuscu’s study which showed increase in the trabecular numbers, in the bone face after using osteotome technique [12].

Nevertheless, in a 2017 study, the survival of autogenous bone grafts taken from rabbit calvaria was evaluated after the grafts were mixed with several antimicrobial agents, including ozone gel, as a study group. They observed better outcomes for the ozone-treated group, which were demonstrated by a significant increase in the creation of new bone and a significantly larger percentage of normal osteocytes. Furthermore, scan revealed that the ozone-treated group's bone mineral density and concentration significantly increased in comparison to the other groups under study [17]. These findings aligned with those of a different 2018 study that shown that ozone gel speeds up the development and maturation of new bone [10].

Another study conducted in 2024 by Ahmed et al. demonstrated that ozone gel improved marginal bone preservation, possibly as a result of osteoblast activation, osteosynthesis, and a decrease in osteoclastic activity. Ozone causes cytokines, particularly Transforming Growth Factor TGF-β1, to be expressed at greater levels. Additionally, TGF-β1 clearly affects angiogenesis, collagen and extracellular matrix formation, and cell proliferation (monocytes and fibroblasts) When compared to group (I) (control group), which received bone graft alone without ozone gel, the study group that employed bone graft combined with ozone gel demonstrated a statistically significant decrease in marginal bone loss at 9 months postoperatively. However, there was no statistically significant difference at(baseline), (3 m) and (6 m) between (Group I) and (Group II) [3]. This explains the disagreement of results with our study as in our study the time interval for bone measurements was four months postoperative. Moreover, in our study ozone gel was used solely without the use of bone graft.

Therefore, the contradiction in our study with the previously mentioned studies can be due to the difference in the form of ozone gel used, as in our study ozone gel was used solely without any scaffold material which may cause fast reabsorption of the ozone gel and this might justify the lack of significant superiority of the ozone gel recipient group in spite of higher statistically significant results in other studies and this will be an interesting point for other future studies.

## Conclusion

Ozone gel has no determinate effects on bone healing around implants inserted simultaneously with internal sinus elevation and offered better results in terms of the bone width and buccal plate of bone thickness as compared to graftless tenting technique.

## Supplementary Information

Below is the link to the electronic supplementary material.

Supplementary file1 (JPG 111 KB)

Supplementary file2 (DOC 222 KB)

Supplementary file3 (PDF 111 KB)

Supplementary file4 (HEIC 258 KB)

Supplementary file5 (HEIC 142 KB)

## Data Availability

The datasets generated during and analyzed during the current study are available from the corresponding author upon request.

## Footnotes

1 Augmentin ®, GlaxoSmithKline, UK.

## Abbreviations

CBCT: Cone beam computed tomography
