# Supplementary figures and images for: Does ozone gel enhance the bone width and buccal plate of bone thickness surrounding the implant following osseodensification? A randomized controlled clinical trial
Source: Oral Maxillofac Surg. 2025 Apr 14;29(1):82. doi: 10.1007/s10006-025-01367-x (PMC11996989; doi:10.1007/s10006-025-01367-x)

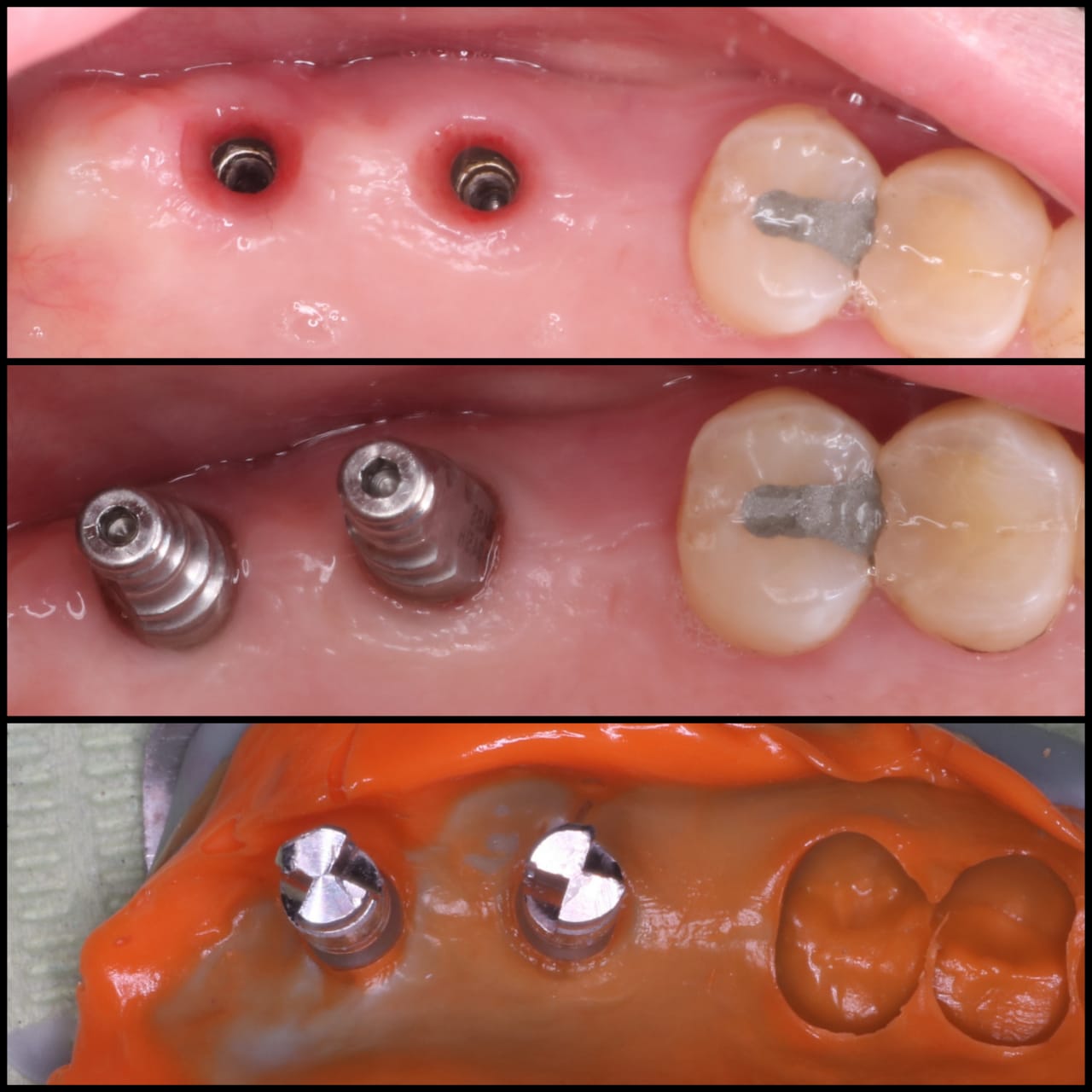

Supplement: Supplementary file 1 — Supplementary file1 (JPG 111 KB) [file 10006_2025_1367_MOESM1_ESM.jpg]
