# Supplementary material for: Does ozone gel enhance the bone width and buccal plate of bone thickness surrounding the implant following osseodensification? A randomized controlled clinical trial
Source: Oral Maxillofac Surg. 2025 Apr 14;29(1):82. doi: 10.1007/s10006-025-01367-x (PMC11996989; doi:10.1007/s10006-025-01367-x)
Supplement: Supplementary file 3 — Supplementary file3 (PDF 111 KB) [file 10006_2025_1367_MOESM3_ESM.pdf]

ClinicalTrials.gov Protocol Registration and Results System (PRS) Receipt
Release Date: September 25, 2024

ClinicalTrials.gov ID: NCT06604819

Study Identification

Unique Protocol ID: 417/2021

Brief Title: The Impact of Ozone Gel on the Bone Height and Density

Official Title: The Impact of Ozone Gel on the Bone Height and Density After Closed Sinus Lifting With Simultaneous Implant Placement: A Randomized Controlled Clinical Trial.

Secondary IDs:

Study Status

Record Verification: September 2024

Overall Status: Completed

Study Start: December 21, 2021 [Actual]

Primary Completion: December 15, 2022 [Actual]

Study Completion: December 20, 2023 [Actual]

Sponsor/Collaborators

Sponsor: Suez Canal University

Responsible Party: Principal Investigator
Investigator: Dina yousry [Dina_yousry]
Official Title: Principle investigator "Doctorate candidate"
Affiliation: Suez Canal University

Collaborators:

Oversight

U.S. FDA-regulated Drug: No

U.S. FDA-regulated Device: No

U.S. FDA IND/IDE: No

Human Subjects Review: Board Status: Approved
Approval Number: 417/2021
Board Name: The Ethics Committee of faculty of dentistry, Suez Canal University
Board Affiliation: Faculty of dentistry, Suez Canal University
Phone: 00201223366424
Email: mohamed_elshlkami@dent.suez.edu.eg
Address:

J7FG+4CW, The Ring Rd, El Sheikh Zayed, Ismailia 3, Ismailia Governorate 8366004

Data Monitoring: Yes

Study Description

Brief Summary: Purpose: The present study was conducted to evaluate the effect of using ozone gel with maxillary sinus elevation using tenting technique on the clinical and radiographic outcome of implants placed simultaneously..

Materials and Methods: A total of 30 sinuses with an average residual alveolar bone height ranging from 4-7 mm participated in this randomized controlled clinical trial. After closed sinus lift operation, patients were randomly and equally allocated into control group and ozone gel recipient group. Cone beam computed tomography was performed immediately and at 4 months postoperatively. Bone stability was measured immediately and at 3 & 4 months postoperatively. Bone height and stability were evaluated radiographically, and bone stability was measured using the Osstell device.

Detailed Description: Concern over biological augmentations used in bone healing has grown in recent years. One of the essential demands in sinus lifting procedure is to speed up the bone formation and enhance the quality of the formed bone in the space created after sinus elevation. Since more than 100 years medical grade ozone has been used as one of the non-medication methods of treatment. E. A. Fisch in the 1930’s was the first dentist to use ozone therapy in his practice to aid in disinfection and wound healing. Current ozone uses a mixture of ozone gel and pure oxygen, with today’s medical ozone generators which regulate the flow of medical grade O2 through high voltage tubes which is capable of producing pure ozone-oxygen mixtures in precise dosages.

Nevertheless, there is not enough evidence to support the use of ozone in oral and maxillofacial surgery. Ozone therapy has a therapeutic effect that promotes blood and growth factor supply, aids in wound healing, and may improve bone regeneration . However up till now, no studies were conducted to assess the impact of using ozone gel on bone formation in case maxillary sinus elevation.

Conditions

Conditions: Sinus Pneumatization

Keywords: closed sinus lifting

Study Design

Study Type: Interventional

Primary Purpose: Treatment

Study Phase: N/A

Interventional Study Model: Parallel Assignment

Number of Arms: 2

Masking: Double (Participant, Outcomes Assessor)
biostatistician

Allocation: Randomized

Enrollment: 30 [Actual]

Arms and Interventions

Arms	Assigned Interventions
Active Comparator: closed sinus lifting using osteotomes with simultaneous implant placement<br>Full thickness mucoperiosteal flap was elevated.• The pilot drill of the implant system was used to create an osteotomy 1 mm short of the subantral floor. • The insertion of the osteotome were repeated several times until the required membrane lift was achieved then the osteotome corresponding in size to the last drill was used.After that, placement of the implant was done .Healing abutment was placed to allow for multiple times measurements of implant stability and primary closure was done and the flap was sutured in an interrupted manner using 4/0 prolene suture .	Procedure/Surgery: closed sinus lifting using osteotomes with simultaneous implant placement<br>The patient received closed sinus lifting using osteotomes with implant placed simultaneously.
Experimental: closed sinus lifting using osteotomes with simultaneous implant placement with ozone gel placement<br>Full thickness mucoperiosteal flap was elevated.• The pilot drill of the implant system was used to create an osteotomy 1 mm short of the subantral floor. • The insertion of the osteotome were repeated several times until the required membrane lift was achieved then the osteotome corresponding in size to the last drill was used.Ozone gel was delivered into the osteotomy . To obtain the ozone gel, pure olive oil	Drug: Ozone gel placed after closed sinus lifting then implant placed simultaneously.<br>The patient received closed sinus lifting using osteotomes then ozone gel is prepared. Pure olive oil was blasted with 25 μ/ml O_3 gas for two days, or until the oil changed from a greenish-colored liquid to a whitish gel. The longevity Ext 120 ozone generator executed out this process then implants were placed.

Arms	Assigned Interventions
was blasted with 25 µ/ml O3 gas for two days, or until the oil changed from a greenish-colored liquid to a whitish gel. The longevity Ext 120 ozone generator executed out this process. After that, placement of the implant was done .Healing abutment was placed to allow for multiple times measurements of implant stability and primary closure was done and the flap was sutured in an interrupted manner using 4/0 prolene suture .	

Outcome Measures

[See Results Section.]

Eligibility

Minimum Age: 18 Years

Maximum Age:

Sex: All

Gender Based: No

Accepts Healthy Volunteers: No

Criteria: Inclusion Criteria:

- Patient age should be > 18 years of age.
- Patients included were ASA I and ASA II.
- Both males and females were included in the study.
- Patients with need of tooth replacements in the maxillary premolar and molar area where the residual alveolar bone height ranges from 4-7 mm and bone density D3 or D4.
- Compliance with all requirements in the study and signing the informed consent.

Exclusion Criteria:

- Patients with immunological diseases or diseases affecting bone healing.
- Patients suffering from uncontrolled systemic diseases.
- Patients with active acute infection related to the planned implant site, maxillary sinusitis or pathosis.
- Patients with parafunctional occlusal habits.
- Patients with bad oral hygiene.
- Patients who were alcohol and drug abusers.
- Heavy Smoker Patients.
- Patients who went previous sinus lifting surgery.

Contacts/Locations

Central Contact Person: Dina Y El-Zaefzaf, Masters
Telephone: 00201222298685
Email: Dina_yousry@dent.suez.edu.eg

Central Contact Backup:

Study Officials: Dina El-Zaefzaf, Masters
Study Principal Investigator
Faculty of Dentistry,Suez Canal University

Locations: **Egypt**
Faculty of Dentistry, Suez Canal University
Ismailia, Egypt
Contact: Dina Y El-Zefzaf, Masters 00201222298685 Dina_Yousry@dent.suez.edu.eg

IPDSharing

Plan to Share IPD: Yes
It will be available upon request

Supporting Information:
Study Protocol
Statistical Analysis Plan (SAP)
Informed Consent Form (ICF)

Time Frame:
It will be available after the research is published.

Access Criteria:
It will be available upon request

URL:

References

Citations:

Links:

Available IPD/Information:

Documents

Study Protocol, Statistical Analysis Plan and Informed Consent Form
Document Date: December 21, 2021
Uploaded: 09/25/2024 05:57

Study Results

Participant Flow

Reporting Groups

	Description
Closed Sinus Lifting Using Osteotomes With Simultaneous Implant Placement	Full thickness mucoperiosteal flap was elevated.• The pilot drill of the implant system was used to create an osteotomy 1 mm short of the subantral floor. • The insertion of the osteotome were repeated several times until the required membrane lift was achieved then the osteotome corresponding in size to the last drill was used.After that, placement of the implant was done .Healing abutment was placed to allow for multiple times measurements of implant stability and primary closure was done and the flap was sutured in an interrupted manner using 4/0 prolene suture .<br><br>closed sinus lifting using osteotomes with simultaneous implant placement: The patient received closed sinus lifting using osteotomes with implant placed simultaneously.
Closed Sinus Lifting Using Osteotomes With Simultaneous Implant Placement With Ozone Gel Placement	Full thickness mucoperiosteal flap was elevated.• The pilot drill of the implant system was used to create an osteotomy 1 mm short of the subantral floor. • The insertion of the osteotome were repeated several times until the required membrane lift was achieved then the osteotome corresponding in size to the last drill was used.Ozone gel was delivered into the osteotomy . To obtain the ozone gel, pure olive oil was blasted with 25 μ/ml O_3 gas for two days, or until the oil changed from a greenish-colored liquid to a whitish gel. The longevity Ext 120 ozone generator executed out this process. After that, placement of the implant was done .Healing abutment was placed to allow for multiple times measurements of implant stability and primary closure was done and the flap was sutured in an interrupted manner using 4/0 prolene suture .<br><br>Ozone gel placed after closed sinus lifting then implant placed simultaneously.: The patient received closed sinus lifting using osteotomes then ozone gel is prepared. Pure olive oil was blasted with 25 μ/ml O_3 gas for two days, or until the oil changed from a greenish-colored liquid to a whitish gel. The longevity Ext 120 ozone generator executed out this process then implants were placed.

Overall Study

	Closed Sinus Lifting Using Osteotomes With Simultaneous Implant Placement	Closed Sinus Lifting Using Osteotomes With Simultaneous Implant Placement With Ozone Gel Placement
Started	15	15
Completed	15	15

	Closed Sinus Lifting Using Osteotomes With Simultaneous Implant Placement	Closed Sinus Lifting Using Osteotomes With Simultaneous Implant Placement With Ozone Gel Placement
Not Completed	0	0

Baseline Characteristics

Reporting Groups

	Description
Closed Sinus Lifting Using Osteotomes With Simultaneous Implant Placement	Full thickness mucoperiosteal flap was elevated.• The pilot drill of the implant system was used to create an osteotomy 1 mm short of the subantral floor. • The insertion of the osteotome were repeated several times until the required membrane lift was achieved then the osteotome corresponding in size to the last drill was used.After that, placement of the implant was done .Healing abutment was placed to allow for multiple times measurements of implant stability and primary closure was done and the flap was sutured in an interrupted manner using 4/0 prolene suture .<br><br>closed sinus lifting using osteotomes with simultaneous implant placement: The patient received closed sinus lifting using osteotomes with implant placed simultaneously.
Closed Sinus Lifting Using Osteotomes With Simultaneous Implant Placement With Ozone Gel Placement	Full thickness mucoperiosteal flap was elevated.• The pilot drill of the implant system was used to create an osteotomy 1 mm short of the subantral floor. • The insertion of the osteotome were repeated several times until the required membrane lift was achieved then the osteotome corresponding in size to the last drill was used.Ozone gel was delivered into the osteotomy . To obtain the ozone gel, pure olive oil was blasted with 25 μ/ml O_3 gas for two days, or until the oil changed from a greenish-colored liquid to a whitish gel. The longevity Ext 120 ozone generator executed out this process. After that, placement of the implant was done .Healing abutment was placed to allow for multiple times measurements of implant stability and primary closure was done and the flap was sutured in an interrupted manner using 4/0 prolene suture .<br><br>Ozone gel placed after closed sinus lifting then implant placed simultaneously.: The patient received closed sinus lifting using osteotomes then ozone gel is prepared. Pure olive oil was blasted with 25 μ/ml O_3 gas for two days, or until the oil changed from a greenish-colored liquid to a whitish gel. The longevity Ext 120 ozone generator executed out this process then implants were placed.

Baseline Measures

		Closed Sinus Lifting Using Osteotomes With Simultaneous Implant Placement	Closed Sinus Lifting Using Osteotomes With Simultaneous Implant Placement With Ozone Gel Placement	Total
Overall Number of Participants		15	15	30
**Age, Categorical**<br>Measure Type: Count of Participants<br>Unit of measure: participants	Number Analyzed	15 participants	15 participants	30 participants
	<=18 years	0 0%	0 0%	0 0%

		Closed Sinus Lifting Using Osteotomes With Simultaneous Implant Placement	Closed Sinus Lifting Using Osteotomes With Simultaneous Implant Placement With Ozone Gel Placement	Total
	Between 18 and 65 years	15 100%	15 100%	30 100%
	>=65 years	0 0%	0 0%	0 0%
**Age, Continuous**<br>Mean (Standard Deviation)<br>Unit of measure: years	Number Analyzed	15 participants	15 participants	30 participants
		43.1 (4.5)	42.9 (4.9)	43 (4.7)
**Sex: Female, Male**<br>Measure Type: Count of Participants<br>Unit of measure: participants	Number Analyzed	15 participants	15 participants	30 participants
	Female	13 86.67%	14 93.33%	27 90%
	Male	2 13.33%	1 6.67%	3 10%
**Race and Ethnicity Not Collected** [1]<br>Measure Type: Count of Participants<br>Unit of measure: participants	Number Analyzed	0 participants	0 participants	0 participants
		---	---	0
		[1] Measure Analysis Population Description: Race and Ethnicity were not collected from any participant.		
**Region of Enrollment**<br>Measure Type: Count of Participants<br>Unit of measure: participants	Number Analyzed	15 participants	15 participants	30 participants
Egypt		15 100%	15 100%	30 100%

Outcome Measures

1. Primary Outcome Measure:

Measure Title	Implant Stability

Measure Description	- Resonance frequency analysis was performed to assess implant stability. The implant stability was measured using the Osstell ISQ scale “immediate postoperative then after 3 and 4 months . The readings were taken along the three surfaces of the implant (perpendicular to the implant axis, mesial surface and distal surface). Finally, the averages of these readings were taken as a representative value for each implant .
Time Frame	Immediate postoperative,3 months and 4 months postoperative

Analysis Population Description
[Not Specified]

Reporting Groups

	Description
Closed Sinus Lifting Using Osteotomes With Simultaneous Implant Placement	Full thickness mucoperiosteal flap was elevated.• The pilot drill of the implant system was used to create an osteotomy 1 mm short of the subantral floor. • The insertion of the osteotome were repeated several times until the required membrane lift was achieved then the osteotome corresponding in size to the last drill was used.After that, placement of the implant was done .Healing abutment was placed to allow for multiple times measurements of implant stability and primary closure was done and the flap was sutured in an interrupted manner using 4/0 prolene suture .<br><br>closed sinus lifting using osteotomes with simultaneous implant placement: The patient received closed sinus lifting using osteotomes with implant placed simultaneously.
Closed Sinus Lifting Using Osteotomes With Simultaneous Implant Placement With Ozone Gel Placement	Full thickness mucoperiosteal flap was elevated.• The pilot drill of the implant system was used to create an osteotomy 1 mm short of the subantral floor. • The insertion of the osteotome were repeated several times until the required membrane lift was achieved then the osteotome corresponding in size to the last drill was used.Ozone gel was delivered into the osteotomy . To obtain the ozone gel, pure olive oil was blasted with 25 μ/ml O3 gas for two days, or until the oil changed from a greenish-colored liquid to a whitish gel. The longevity Ext 120 ozone generator executed out this process. After that, placement of the implant was done .Healing abutment was placed to allow for multiple times measurements of implant stability and primary closure was done and the flap was sutured in an interrupted manner using 4/0 prolene suture .<br><br>Ozone gel placed after closed sinus lifting then implant placed simultaneously.: The patient received closed sinus lifting using osteotomes then ozone gel is prepared. Pure olive oil was blasted with 25 μ/ml O3 gas for two days, or until the oil changed from a greenish-colored liquid to a whitish gel. The longevity Ext 120 ozone generator executed out this process then implants were placed.

Measured Values

	Closed Sinus Lifting Using Osteotomes With Simultaneous Implant Placement	Closed Sinus Lifting Using Osteotomes With Simultaneous Implant Placement With Ozone Gel Placement
Overall Number of Participants Analyzed	15	15

	Closed Sinus Lifting Using Osteotomes With Simultaneous Implant Placement	Closed Sinus Lifting Using Osteotomes With Simultaneous Implant Placement With Ozone Gel Placement
Implant Stability<br>Mean (Standard Deviation)<br>Unit of measure: units on a scale	84 (3.7)	86.7 (6.7)

2. Secondary Outcome Measure:

Measure Title	Bone Density
Measure Description	The bone density was evaluated using CBCT. To minimize the error from radiographic image alignment, the On Demand 3D fusion module software was used to generate a superimposed image at the sagittal and coronal planes of each implant to compare each parameter .
Time Frame	Immediate postoperative and 4 months postoperative

Analysis Population Description
[Not Specified]

Reporting Groups

	Description
Closed Sinus Lifting Using Osteotomes With Simultaneous Implant Placement	Full thickness mucoperiosteal flap was elevated.• The pilot drill of the implant system was used to create an osteotomy 1 mm short of the subantral floor. • The insertion of the osteotome were repeated several times until the required membrane lift was achieved then the osteotome corresponding in size to the last drill was used.After that, placement of the implant was done .Healing abutment was placed to allow for multiple times measurements of implant stability and primary closure was done and the flap was sutured in an interrupted manner using 4/0 prolene suture .<br><br>closed sinus lifting using osteotomes with simultaneous implant placement: The patient received closed sinus lifting using osteotomes with implant placed simultaneously.

	Description
Closed Sinus Lifting Using Osteotomes With Simultaneous Implant Placement With Ozone Gel Placement	Full thickness mucoperiosteal flap was elevated.• The pilot drill of the implant system was used to create an osteotomy 1 mm short of the subantral floor. • The insertion of the osteotome were repeated several times until the required membrane lift was achieved then the osteotome corresponding in size to the last drill was used.Ozone gel was delivered into the osteotomy . To obtain the ozone gel, pure olive oil was blasted with 25 μ/ml O3 gas for two days, or until the oil changed from a greenish-colored liquid to a whitish gel. The longevity Ext 120 ozone generator executed out this process. After that, placement of the implant was done .Healing abutment was placed to allow for multiple times measurements of implant stability and primary closure was done and the flap was sutured in an interrupted manner using 4/0 prolene suture .<br><br>Ozone gel placed after closed sinus lifting then implant placed simultaneously.: The patient received closed sinus lifting using osteotomes then ozone gel is prepared. Pure olive oil was blasted with 25 μ/ml O3 gas for two days, or until the oil changed from a greenish-colored liquid to a whitish gel. The longevity Ext 120 ozone generator executed out this process then implants were placed.

Measured Values

	Closed Sinus Lifting Using Osteotomes With Simultaneous Implant Placement	Closed Sinus Lifting Using Osteotomes With Simultaneous Implant Placement With Ozone Gel Placement
Overall Number of Participants Analyzed	15	15
Bone Density<br>Mean (Standard Deviation)<br>Unit of measure: units on a scale	272.3 (119.1)	363.6 (178.6)

3. Secondary Outcome Measure:

Measure Title	Bone Height Gain
Measure Description	Cone beam computed tomography (CBCT was taken to evaluate the accuracy of implant placement and to represent the baseline measurement for evaluation. The bone height gain was evaluated along the surface of each implant.
Time Frame	Immediate postoperative and 4 months postoperative

Analysis Population Description

[Not Specified]

Reporting Groups

	Description
Closed Sinus Lifting Using Osteotomes With Simultaneous Implant Placement	Full thickness mucoperiosteal flap was elevated.• The pilot drill of the implant system was used to create an osteotomy 1 mm short of the subantral floor. • The insertion of the osteotome were repeated several times until the required membrane lift was achieved then the osteotome corresponding in size to the last drill was used.After that, placement of the implant was done .Healing abutment was placed to allow for multiple times measurements of implant stability and primary closure was done and the flap was sutured in an interrupted manner using 4/0 prolene suture .<br><br>closed sinus lifting using osteotomes with simultaneous implant placement: The patient received closed sinus lifting using osteotomes with implant placed simultaneously.
Closed Sinus Lifting Using Osteotomes With Simultaneous Implant Placement With Ozone Gel Placement	Full thickness mucoperiosteal flap was elevated.• The pilot drill of the implant system was used to create an osteotomy 1 mm short of the subantral floor. • The insertion of the osteotome were repeated several times until the required membrane lift was achieved then the osteotome corresponding in size to the last drill was used.Ozone gel was delivered into the osteotomy . To obtain the ozone gel, pure olive oil was blasted with 25 μ/ml O3 gas for two days, or until the oil changed from a greenish-colored liquid to a whitish gel. The longevity Ext 120 ozone generator executed out this process. After that, placement of the implant was done .Healing abutment was placed to allow for multiple times measurements of implant stability and primary closure was done and the flap was sutured in an interrupted manner using 4/0 prolene suture .<br><br>Ozone gel placed after closed sinus lifting then implant placed simultaneously.: The patient received closed sinus lifting using osteotomes then ozone gel is prepared. Pure olive oil was blasted with 25 μ/ml O3 gas for two days, or until the oil changed from a greenish-colored liquid to a whitish gel. The longevity Ext 120 ozone generator executed out this process then implants were placed.

Measured Values

	Closed Sinus Lifting Using Osteotomes With Simultaneous Implant Placement	Closed Sinus Lifting Using Osteotomes With Simultaneous Implant Placement With Ozone Gel Placement
Overall Number of Participants Analyzed	15	15
Bone Height Gain<br>Mean (Standard Deviation)<br>Unit of measure: units on a scale	11.09 (0.86)	10.73 (0.65)

Reported Adverse Events

Time Frame	1 year

Adverse Event Reporting Description	No adverse events were detected in all participants.

Reporting Groups

	Description
Closed Sinus Lifting Using Osteotomes With Simultaneous Implant Placement	Full thickness mucoperiosteal flap was elevated.• The pilot drill of the implant system was used to create an osteotomy 1 mm short of the subantral floor. • The insertion of the osteotome were repeated several times until the required membrane lift was achieved then the osteotome corresponding in size to the last drill was used.After that, placement of the implant was done .Healing abutment was placed to allow for multiple times measurements of implant stability and primary closure was done and the flap was sutured in an interrupted manner using 4/0 prolene suture .<br><br>closed sinus lifting using osteotomes with simultaneous implant placement: The patient received closed sinus lifting using osteotomes with implant placed simultaneously.
Closed Sinus Lifting Using Osteotomes With Simultaneous Implant Placement With Ozone Gel Placement	Full thickness mucoperiosteal flap was elevated.• The pilot drill of the implant system was used to create an osteotomy 1 mm short of the subantral floor. • The insertion of the osteotome were repeated several times until the required membrane lift was achieved then the osteotome corresponding in size to the last drill was used.Ozone gel was delivered into the osteotomy . To obtain the ozone gel, pure olive oil was blasted with 25 μ/ml O3 gas for two days, or until the oil changed from a greenish-colored liquid to a whitish gel. The longevity Ext 120 ozone generator executed out this process. After that, placement of the implant was done .Healing abutment was placed to allow for multiple times measurements of implant stability and primary closure was done and the flap was sutured in an interrupted manner using 4/0 prolene suture .<br><br>Ozone gel placed after closed sinus lifting then implant placed simultaneously.: The patient received closed sinus lifting using osteotomes then ozone gel is prepared. Pure olive oil was blasted with 25 μ/ml O3 gas for two days, or until the oil changed from a greenish-colored liquid to a whitish gel. The longevity Ext 120 ozone generator executed out this process then implants were placed.

All-Cause Mortality

	Closed Sinus Lifting Using Osteotomes With Simultaneous Implant Placement	Closed Sinus Lifting Using Osteotomes With Simultaneous Implant Placement With Ozone Gel Placement
	Affected/At Risk (%)	Affected/At Risk (%)
Total All-Cause Mortality	0/0	0/0

Serious Adverse Events

	Closed Sinus Lifting Using Osteotomes With Simultaneous Implant Placement	Closed Sinus Lifting Using Osteotomes With Simultaneous Implant Placement With Ozone Gel Placement
	Affected/At Risk (%)	Affected/At Risk (%)
Total	0/0	0/0

Other Adverse Events

Frequency Threshold Above Which Other Adverse Events are Reported: 0%

	Closed Sinus Lifting Using Osteotomes With Simultaneous Implant Placement	Closed Sinus Lifting Using Osteotomes With Simultaneous Implant Placement With Ozone Gel Placement
	Affected/At Risk (%)	Affected/At Risk (%)
Total	0/0	0/0

Limitations and Caveats

[Not specified]

More Information

Certain Agreements:

Principal Investigators are NOT employed by the organization sponsoring the study.

There is NOT an agreement between the Principal Investigator and the Sponsor (or its agents) that restricts the PI's rights to discuss or publish trial results after the trial is completed.

Results Point of Contact:

Name/Official Title: Dina Yousry Fouad "Principal investigator"
Organization: Faculty of Dentistry ,Suez Canal University
Phone: 00201222298685
Email: dina_yousry@dent.suez.edu.eg
